# The Eye as a Window to Neurodegeneration: Oxidative Stress, Optic Nerve Vulnerability, and Retinal Biomarkers—A Scoping Review

**DOI:** 10.3390/antiox15080948

**Published:** 2026-07-30

**Authors:** Giustino Varrassi, Y Van Tran, Giacomo Farì, Miguel Narvaez Encinas, Alberto Corriero, Filomena Puntillo, Phong Van Pham, Matteo Luigi Giuseppe Leoni

**Affiliations:** 1 Visionary International Board for Research and Analgesia (VIBRA), Fondazione Paolo Procacci, 00193 Rome, Italy; giuvarr@gmail.com (G.V.); giacomo.fari@unisalento.it (G.F.); miguelandresne@gmail.com (M.N.E.); pbeiu25005@phd.hcmiu.edu.vn (P.V.P.); matteoluigigiuseppe.leoni@uniroma1.it (M.L.G.L.); 2 College of Medicine, University of Baghdad, Baghdad 12114, Iraq; 3 Department of Anesthesiology, Tam Anh General Hospital, Ho Chi Minh City 70000, Vietnam; 4 Lab of Tissue Engineering and Regenerative Medicines, School of Biomedical Engineering, International University, VNU-HCM, Ho Chi Minh City 70000, Vietnam; 5 Department of Experimental Medicine (Di.Me.S.), University of Salento, 73100 Lecce, Italy; 6 Medical Surgeon, University of San Andres, 8390 Calacoto 21 Street, La Paz 202010, Bolivia; 7 Department of Emergency and Organ Transplants, University of Bari, 70121 Bari, Italy; alberto.corriero@gmail.com (A.C.); filomena.puntillo@uniba.it (F.P.); 8 Department of Medical and Surgical Sciences and Translational Medicine, Sapienza University of Rome, 00185 Rome, Italy

**Keywords:** neurodegenerative diseases, oxidative stress, retina, optic nerve diseases, retinal ganglion cells, mitochondria, neuroinflammation, biomarkers, glaucoma, precision medicine

## Abstract

Neurodegenerative diseases represent a major and growing global health burden characterized by progressive neuronal dysfunction, axonal degeneration, and irreversible neural tissue loss. Increasing evidence identifies oxidative stress as one of several interacting pathogenic mechanisms in Alzheimer’s disease, Parkinson’s disease, amyotrophic lateral sclerosis, and several optic neuropathies. Interest has increasingly focused on the brain-retina axis, as the retina and optic nerve share structural, metabolic, and molecular features with the central nervous system and may provide accessible insights into neurodegeneration. This scoping review mapped current evidence on oxidative stress in neurodegeneration, emphasizing cranial nerve involvement, optic nerve vulnerability, retinal ganglion cell degeneration, visual dysfunction, oxidative biomarkers, and emerging therapeutic strategies. The review followed established methodological frameworks and PRISMA-ScR recommendations; no formal risk-of-bias appraisal was undertaken, consistent with scoping-review methodology. The literature shows that oxidative stress interacts with mitochondrial dysfunction, neuroinflammation, impaired mitophagy, ferroptosis, and altered bioenergetics, contributing to neuronal injury in cerebral and retinal disorders. Retinal ganglion cells appear particularly vulnerable because of their high metabolic demands and reliance on oxidative phosphorylation. Glaucoma and other optic neuropathies share molecular signatures with central neurodegenerative diseases. Retinal imaging and oxidative biomarkers show promise for diagnosis, monitoring, and stratification. The evidence base is nonetheless dominated by preclinical work; biomarker performance is inconsistent across matrices and assay platforms and most antioxidant clinical trials have been negative. Oxidative stress is therefore best regarded as one interacting node of a broader pathogenic network rather than a universal or predominant driver and the brain-retina continuum as a mechanistically plausible but not yet clinically validated framework for biomarker-guided neuroprotection.

## 1. Introduction

Neurodegenerative diseases represent one of the greatest biomedical and public health challenges of the twenty-first century. Population ageing has been accompanied by a marked increase in disorders characterized by progressive neuronal dysfunction and irreversible neural tissue loss, including Alzheimer’s disease (AD), Parkinson’s disease (PD), amyotrophic lateral sclerosis (ALS), Huntington’s disease (HD), multiple sclerosis (MS), and several optic neuropathies [1]. Despite their diverse clinical manifestations and pathological hallmarks, these disorders share convergent molecular mechanisms, among which oxidative stress is one of the most consistently implicated [2].

Oxidative stress results from an imbalance between reactive oxygen and nitrogen species (ROS, RNS) and endogenous antioxidant defences, causing lipid peroxidation, protein oxidation, DNA damage, mitochondrial dysfunction and neuroinflammation [3,4]. The central nervous system (CNS) is particularly vulnerable because of its high oxygen consumption, abundant polyunsaturated fatty acids, intense mitochondrial activity, and limited antioxidant reserves [5]. Current evidence indicates that oxidative stress is both a driver and a consequence of neurodegeneration, participating in self-reinforcing pathogenic loops involving mitochondrial dysfunction, protein aggregation, ferroptosis, impaired autophagy, and chronic neuroinflammation [6,7,8].

Within these loops, activated glia act as reciprocal amplifiers of redox injury [9], alongside impaired mitochondrial bioenergetics and defective mitophagy [10,11]. These interlocking mechanisms have motivated the search for biomarkers of neurodegenerative activity that precede irreversible clinical change. A recognizable panel of oxidative readouts has been proposed and is set out in Section 3.7 [12,13]. However, methodological heterogeneity and limited standardization continue to restrict their routine application [14].

Cranial nerves may be particularly vulnerable targets of redox-mediated injury [15]. Their long axonal projections, high energetic demands and limited regenerative capacity increase susceptibility to cumulative oxidative damage [16]. Several cranial neuropathies share pathological features with classical neurodegenerative diseases [17].

Among cranial nerves, the optic nerve has attracted particular attention as an accessible model of neurodegeneration: embryologically derived from the diencephalon, it is an anatomical extension of the CNS and shares its structural and molecular architecture [18]. Consequently, degeneration of retinal ganglion cells (RGCs) and their axons is increasingly viewed within the broader framework of CNS neurodegeneration [19,20,21].

The visual system has exceptionally high metabolic demands and RGCs depend on oxidative phosphorylation to sustain axonal transport and synaptic transmission [22]. This dependence renders them vulnerable to redox imbalance, mitochondrial and mitophagic failure, and altered nicotinamide adenine dinucleotide (NAD^+^) metabolism, mechanisms also implicated in AD, PD and ALS [21].

Glaucoma represents the best-characterized model of oxidative stress-related optic neurodegeneration [23,24]. It is now recognized as a complex neurodegeneration involving progressive RGC loss, glial activation, vascular dysregulation and chronic oxidative injury [23,25]. Redox imbalance contributes to RGC apoptosis, lamina cribrosa remodelling and neuroinflammatory responses that drive visual loss [22,26]. Similar mechanisms have been implicated in ischemic optic neuropathy, hereditary mitochondrial optic neuropathies, diabetic retinal neurodegeneration and age-related retinal dysfunction [19,25,27,28,29,30,31]. Retinal and optic nerve biomarkers may in turn report on early neurodegenerative change elsewhere in the CNS [32], supporting the proposal of the retina as a “window to the brain” [18,20].

Despite rapid advances, the interplay among oxidative stress, optic nerve damage and retinal degeneration remains uncertain. This scoping review aims to map contemporary evidence on oxidative stress across neurodegenerative disorders, emphasizing optic nerve vulnerability, RGC degeneration and retinal biomarkers. By integrating evidence across the brain-retina continuum, the review examines how far a shared mechanistic framework linking oxidative stress, mitochondrial dysfunction, neuroinflammation, regulated cell death and retinal imaging biomarkers is actually supported by the available data, and identifies priorities for future translational research. It also weighs the consistency of the evidence, gives explicit space to negative and contradictory findings, and delineates where the “eye as a window” concept is supported and where it remains conjectural.

## 2. Materials and Methods

### 2.1. Study Design

This scoping review was designed to provide a structured overview of the available literature on oxidative stress-related mechanisms across neurodegenerative and neuro-ophthalmological conditions, integrating evidence from central nervous system disorders, cranial nerve pathology, optic nerve involvement, retinal degeneration, and visual dysfunction. The objective was to map an extremely heterogeneous body of mechanistic, translational, imaging, experimental and clinical literature rather than evaluate intervention efficacy. Consequently, a scoping review represented the most appropriate methodology.

The review methodology was developed in accordance with the seminal framework for scoping studies proposed by Arksey et al. [33], subsequently refined by Levac et al. [34], and further guided by the recommendations of the Joanna Briggs Institute (JBI) Manual for Evidence Synthesis [35]. Reporting was performed according to the Preferred Reporting Items for Systematic Reviews and Meta-Analyses Extension for Scoping Reviews (PRISMA-ScR) checklist [36].

The objective of the review was not to evaluate treatment efficacy or perform quantitative synthesis, but rather to comprehensively characterize the breadth, nature, and distribution of available evidence, identify key mechanistic pathways, summarize current biomarkers and therapeutic targets, and highlight existing knowledge gaps requiring future investigation.

### 2.2. Review Question

The review was guided by the following overarching research question:

“What is the current state of evidence regarding the role of oxidative stress in neurodegenerative processes affecting the central nervous system, cranial nerves, optic nerve, retina and visual function?”

To ensure a structured approach, the review question was formulated according to the Population-Concept-Context (PCC) framework recommended by the Joanna Briggs Institute [37].

- Population: Human subjects, experimental animal models, ex vivo tissues, and in vitro systems investigating neurodegenerative processes.
- Concept: Oxidative stress, redox imbalance, ROS, RNS, mitochondrial dysfunction, neuroinflammation, ferroptosis, oxidative biomarkers, and antioxidant therapeutic strategies.
- Context: Neurodegenerative diseases affecting the central nervous system, cranial nerves, optic nerve, retina, and visual pathways.

### 2.3. Eligibility Criteria

Eligibility criteria were defined a priori to identify literature relevant to the role of oxidative stress in neurodegenerative disorders. Particular interest was given to evidence addressing mitochondrial dysfunction, neuroinflammation, RGCs, optic neuropathies and visual impairment within the broader neurodegenerative spectrum.

To provide a comprehensive overview, multiple study designs were included: original research articles, systematic reviews, meta-analyses, scoping reviews, narrative reviews, consensus statements, and clinical practice guidelines. Both preclinical and clinical evidence were considered, including human studies, animal models, translational investigations and in vitro experiments. Only articles published in English between 1 January 2020 and 1 June 2026, were included to capture contemporary developments in the field. This window captures contemporary developments but necessarily excludes the foundational literature on which the field rests, the recognition that the CNS is intrinsically redox-vulnerable and inadequately defended [38], that mitochondrial dysfunction and oxidative stress are jointly implicated across the classical proteinopathies [39], that oxidative stress is a disruption of redox signalling and control rather than a simple excess of oxidants [40] and the identification of the KEAP1-NRF2-ARE axis as the principal inducible defence [41], together with the definition of ferroptosis [42] and the pre-2020 reframing of glaucoma as a neurodegeneration [43,44]. It also excludes almost the entire negative antioxidant trial record (Section 3.10). Landmark work of this kind is cited where it is needed for interpretation, and is identified as such; it was not charted, and it does not enter the counts reported in the Results. The consequences of this asymmetry are considered in Section 4.4.

Because the objective of the review was to map conceptual, mechanistic, translational, and clinical developments across a rapidly evolving multidisciplinary field, review articles were retained and charted separately from primary studies. This approach enabled the identification of emerging themes, research trends, areas of convergence, and persisting knowledge gaps that might not be apparent from individual studies alone.

Review articles were retained because they play a central role in organizing this multidisciplinary field and in propagating the brain–retina analogy. However, reviews and primary studies were treated as non-independent sources, since repeated citation of the same datasets may create an appearance of consensus without true replication. Consequently, no substantive conclusion was based on reviews alone; primary and review-derived evidence were charted separately, and consistency was interpreted qualitatively rather than numerically. Because narrative reviews may also preferentially cite positive findings, null, negative and contradictory evidence was examined in a dedicated section to prevent it from being obscured within the broader mechanistic synthesis. Studies were excluded if they lacked substantive scientific data relevant to the review objectives. Conference abstracts without full-text availability, editorials, commentaries, correspondence and opinion papers were not considered. Publications unrelated to oxidative stress, redox biology, or neurodegenerative mechanisms were excluded, as were studies focused exclusively on acute ocular trauma, infectious diseases, or neoplastic conditions without a neurodegenerative component.

### 2.4. Information Sources

To ensure broad coverage of both biomedical and interdisciplinary literature, four major electronic databases were searched: PubMed/MEDLINE, Embase, Scopus and the Web of Science Core Collection. These databases were selected because of their extensive indexing of clinical, translational, experimental, and basic science research across the fields of neuroscience, ophthalmology, neurobiology and redox biology.

To enhance the sensitivity of the search strategy and minimize the risk of missing relevant publications, additional search techniques were employed. The reference lists of all included studies, as well as those of pertinent review articles, were manually examined to identify further eligible records. Forward and backward citation tracking was also performed to capture influential studies that may not have been retrieved through the primary database searches. This multi-source approach was adopted to maximize the comprehensiveness of the evidence base and ensure the inclusion of the most relevant and up-to-date literature available on the topic.

### 2.5. Search Strategy

The search strategy was designed to achieve a comprehensive retrieval of the literature pertaining to oxidative stress and its role in neurodegeneration, with particular focus on optic nerve pathology, retinal neurodegeneration, and visual dysfunction. In accordance with recommendations from the Joanna Briggs Institute [35], the strategy combined controlled vocabulary terms, including Medical Subject Headings (MeSH) and Emtree descriptors, with free-text keywords to maximize both sensitivity and specificity across the selected databases.

Search terms were developed iteratively around three core domains: oxidative stress and redox biology; neurodegenerative diseases; and ocular, retinal, and optic nerve involvement. The oxidative stress domain included terms such as “oxidative stress”, “reactive oxygen species”, “ROS”, “reactive nitrogen species”, “RNS”, “redox”, “ferroptosis” and “mitochondrial dysfunction”. These were combined with neurodegeneration-related terms including “neurodegeneration”, “Alzheimer disease”, “Parkinson disease”, “amyotrophic lateral sclerosis”, “ALS”, “Huntington disease” and “dementia”. To capture visual system involvement, additional terms included “optic nerve”, “retina”, “retinal ganglion cell”, “glaucoma”, “optic neuropathy”, “vision”, “visual dysfunction” and “neuro-ophthalmology”.

For PubMed/MEDLINE, these concepts were combined using Boolean operators to identify relevant experimental and clinical studies. Equivalent search strategies were adapted for Embase, Scopus, and Web of Science according to their indexing systems, syntax requirements, and controlled vocabularies. Database-specific filters and indexing terms were used when appropriate to optimize retrieval while maintaining methodological consistency. The complete search strategies are provided in the Supplemental Table S1.

The strategy was refined through pilot searches and iterative testing against key known publications. The final search was designed to maximize comprehensiveness and sensitivity while maintaining sufficient precision to minimize retrieval of irrelevant records.

### 2.6. Study Selection

All records identified through database and supplementary searches were imported into Zotero 9.0 (Corporation for Digital Scholarship, Vienna, VA, USA), and duplicate entries were removed before screening. Study selection was conducted in two sequential phases. First, titles and abstracts were screened to identify potentially relevant publications. Second, complete manuscripts were assessed against the predefined eligibility criteria to determine final inclusion.

To ensure methodological rigour and reduce selection bias, screening and eligibility assessments were performed independently by three reviewers (YVT, PVP, and MNE). Disagreements regarding study inclusion were resolved through discussion and consensus. When consensus could not be achieved, a fourth reviewer (MLGL) provided an independent evaluation and final decision. Reasons for exclusion at the full-text stage were systematically recorded to enhance transparency and reproducibility.

The overall process of study identification, screening, eligibility assessment and inclusion was documented using a PRISMA-ScR flow diagram, in accordance with current recommendations for reporting scoping reviews.

### 2.7. Data Extraction

Data extraction was conducted using a standardized charting form built in Microsoft Excel (Microsoft 365; Microsoft Corp., Redmond, WA, USA) and developed according to recommendations for scoping reviews. The form ensured systematic and consistent data collection while accommodating the diversity of evidence relevant to the review objectives. For each included study, information was extracted on bibliographic and methodological characteristics, including authors, year of publication, country, study design and population or experimental model. Particular attention was given to the neurodegenerative condition investigated, ocular or visual system involvement and the oxidative stress mechanisms examined.

Additional data included molecular, biochemical, imaging and functional biomarkers of oxidative damage, neurodegeneration, or retinal dysfunction. Information on therapeutic targets, pharmacological interventions, neuroprotective strategies, and other translational approaches was also recorded when available. The principal findings of each study were summarized, together with methodological limitations, research priorities, and knowledge gaps identified by the authors.

To ensure accuracy and reproducibility, data extraction was performed independently by the same three reviewers. Completed charting forms were cross-checked and verified, and any discrepancies were resolved through discussion and consensus. This process minimized extraction errors and ensured a reliable synthesis of the available evidence.

### 2.8. Data Synthesis and Presentation of Results

In accordance with the methodological principles supporting scoping reviews, the present study was designed to provide a comprehensive mapping of the available evidence rather than to generate pooled estimates of effect or determine the comparative effectiveness of specific interventions. Consistent with current Joanna Briggs Institute guidance and PRISMA-ScR recommendations, a formal risk-of-bias assessment and quantitative meta-analysis were not undertaken, as the primary objective of the review was to characterize the breadth, diversity and nature of the existing literature across a complex and heterogeneous field of research. This approach is considered appropriate when the purpose of the evidence synthesis is exploratory and descriptive, particularly in areas characterized by diverse study designs, experimental models, mechanistic investigations, and emerging translational evidence.

Following data extraction, the included studies were synthesized descriptively through evidence mapping and thematic analysis. Primary studies and review articles were considered complementary sources of evidence. While primary studies informed mechanistic, experimental, and clinical findings, review articles facilitated the identification of overarching conceptual frameworks, emerging research directions, and cross-disciplinary areas of convergence. Findings were presented using summary tables, evidence matrices, thematic classifications and graphical representations of key biological and mechanistic pathways. This approach provided a comprehensive overview of the literature while illustrating relationships among oxidative stress, neurodegenerative mechanisms, retinal pathology, and visual dysfunction.

To ensure conceptual consistency, the evidence was organized into eight predefined thematic domains: (1) oxidative stress and central nervous system neurodegeneration; (2) mitochondrial dysfunction and redox imbalance; (3) neuroinflammation and oxidative signalling; (4) cranial nerve degeneration; (5) optic nerve and retinal neurodegeneration; (6) oxidative stress biomarkers; (7) the retina as a biomarker of CNS disease; and (8) therapeutic, neuroprotective, and translational implications.

This framework enabled the integration of findings from clinical studies, experimental models, translational investigations, and review literature while preserving the methodological diversity of the field. Beyond summarizing current knowledge, the synthesis identified areas of convergence across research domains, highlighted emerging mechanistic and translational trends, and delineated persistent knowledge gaps that may guide future experimental and clinical investigations.

## 3. Results

The database searches identified 2121 records. Records were retrieved from PubMed/MEDLINE (*n* = 812), Embase (*n* = 486), Scopus (*n* = 515) and the Web of Science Core Collection (*n* = 308). After removal of 1217 duplicates, 904 records underwent title and abstract screening, of which 665 were excluded. Of the 239 reports sought for retrieval, 3 could not be obtained, leaving 236 full-text articles that were assessed for eligibility. Following full-text review, 61 reports were excluded because they lacked an oxidative stress-related mechanism (*n* = 22), a neurodegenerative focus (*n* = 19), or relevant retinal, optic nerve or neuro-ophthalmological outcomes (*n* = 20), yielding 175 included studies. The 175 included studies comprised different types of evidence. Reviews represented the largest component of the literature, with 69 articles, followed by animal studies and in vitro studies, which accounted for 52 and 46 records, respectively. Clinical evidence was limited, with only 6 clinical studies identified, while 1 in silico study and 1 systematic review were also included. Regarding the disease areas addressed, glaucoma was the most frequently investigated condition, with 53 studies, followed by diabetic retinopathy in 30 studies. Broader neurodegenerative diseases were examined in 24 studies, whereas age-related macular degeneration was represented in 13 studies.

**Figure 1:**
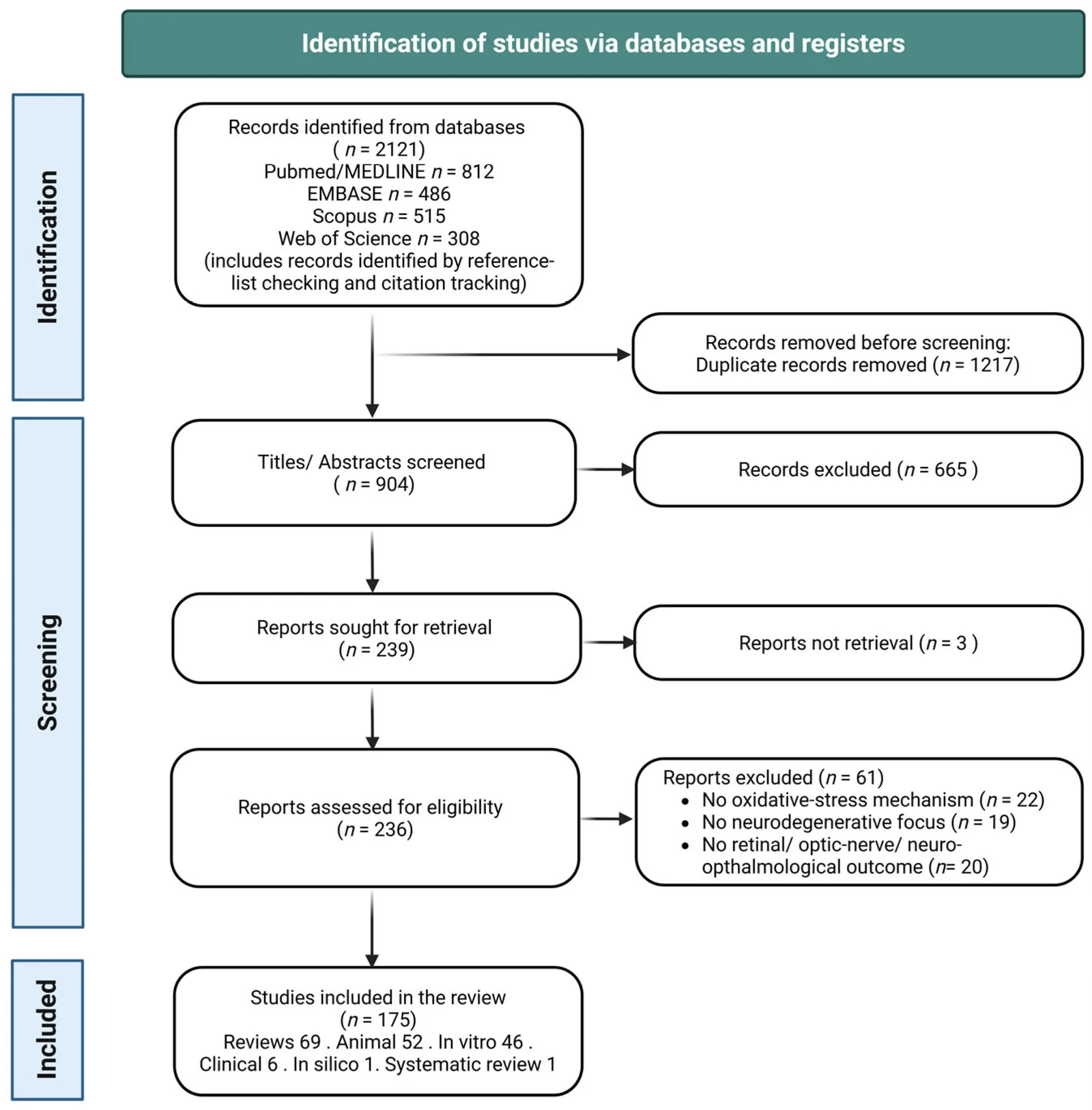
PRISMA-ScR flow diagram for the scoping review. *Created in BioRender. TRAN VAN, Y. (2026) https://BioRender.com/3qt2l04*.

The study identification and selection process is summarized in the PRISMA-ScR flow diagram (Figure 1).

### 3.1. Overview of the Literature

Across the corpus retained for synthesis, oxidative stress emerged not as a peripheral epiphenomenon but as the mechanistic hub around which the contemporary understanding of neurodegeneration is increasingly organized. The mapped literature spanned the full translational continuum (from cell-free redox chemistry and cultured neuroretinal models to transgenic animals, human biofluid studies, and clinical neuro-imaging cohorts) yet converged on a strikingly compact set of recurring motifs: mitochondrial bioenergetic failure, redox-sensitive inflammatory signalling, dysregulated programmed cell death (apoptosis, ferroptosis, pyroptosis and their hybrids), defective autophagy and mitophagy and progressive axonal attrition [16,45,46,47]. A second organizing observation was anatomical. The retina and optic nerve recurred throughout the dataset as the neural compartments in which these motifs could be observed earliest, quantified most precisely and perturbed most readily, by virtue of their exceptional metabolic load, mitochondrial density and surgical accessibility [45,48,49]. Representative studies spanning these mechanistic, anatomical and translational themes are summarized in Table 1.

### 3.2. Domain 1. Oxidative Stress and Central Nervous System Neurodegeneration

The pathogenic centrality of oxidative stress in the canonical neurodegenerative diseases was the most uniformly reported finding. Syntheses addressing AD, PD, ALS and HD described a shared sequence in which excess ROS/RNS overwhelm glutathione- and thioredoxin-based defences, producing lipid peroxidation, protein carbonylation, nucleic-acid oxidation and ultimately synaptic failure and neuronal apoptosis [7,62,63,64]. Mitochondrial dysfunction emerged as a shared mechanistic substrate linking the four classical proteinopathies, a convergence that is also reflected in models of dementia comorbidity [12,62,65,66].

A second, near-unanimous theme was directional: redox imbalance was reframed from a terminal by-product of dying neurons into an upstream initiator and feed-forward amplifier of degeneration [2,67,68]. The strongest form of this claim, mutual potentiation between oxidative stress and neuroinflammation, was documented in dopaminergic and demyelinating disease alike [11,69]. Additionally, the principle generalized beyond the common diseases to orphan neurodegenerative conditions and to the ocular manifestations of systemic disease, where dopaminergic, α-synuclein- and mitochondria-related abnormalities were detected in parkinsonian eyes [70,71]. Notably, no study positioned oxidative stress as merely epiphenomenal, yet the reviews consistently situated it within, rather than above, an interacting pathogenic network.

### 3.3. Domain 2. Mitochondrial Dysfunction and Redox Imbalance

If oxidative stress was the most frequently invoked mechanism, mitochondrial dysfunction was its most frequently invoked source and amplifier. Studies across cerebral and retinal models implicated a shared lesion set (impaired oxidative phosphorylation, electron-transport-chain leak, collapse of membrane potential, mitochondrial DNA damage, and disturbed fission-fusion dynamics) simultaneously as generators of pathological ROS and as their casualties [8,45,47,72,73,74,75]. Within this set, mitochondrial dynamics emerged as a recurrent node of vulnerability, with Drp1-mediated fission linking pathologically elevated intraocular pressure to ganglion-cell death [50].

Quality-control machinery featured prominently. Axonal mitophagy emerged as a determinant of ganglion-cell fate, with mitophagic failure specifically implicated in glaucomatous degeneration and threshold-dependent ROS handling proposed as a “precision therapeutic window” [47,76,77]. The corollary (that augmenting mitochondrial competence is protective) was supported experimentally. Several studies demonstrated that boosting neuronal mitochondrial activity limits neurodegeneration in a murine model of MS by enhancing mitochondrial biogenesis in stem-cell-derived ganglion cells, augmenting neuronal mitochondrial activity in demyelinating disease [52,55]. Restoration of NAD^+^ homeostasis was a frequent therapeutic correlate of these findings [78,79].

Underlying the domain was a consistent anatomical rationale. The RGC was repeatedly singled out as a sentinel of mitochondrial integrity. Its long, initially unmyelinated intraocular axon and high tonic firing impose an energetic burden that leaves little reserve against bioenergetic insult, which is a vulnerability made concrete by the localization of retinal DNA and RNA oxidation to ganglion-cell mitochondria [22,45,49]. Inherited mitochondrial optic neuropathies, including Leber hereditary optic neuropathy (LHON) and dominant optic atrophy, further illustrated this principle as natural disease models of RGC susceptibility [80]. The reported co-segregation of ALS with LHON suggested that mitochondrial dysfunction may act as a shared disease modifier across motor and visual phenotypes, while broader ocular evidence consolidated mitochondrial failure as a convergent pathogenic mechanism across retinal and optic nerve disease [27,45,54].

### 3.4. Domain 3. Neuroinflammation and Oxidative Signalling

Neuroinflammation and oxidative signalling emerged as a closely coupled pathogenetic axis, rather than as separate processes [12,26,46,63]. Microglia, astrocytes, Müller glia and infiltrating myeloid cells were identified as both sensors and amplifiers of oxidative injury, generating ROS, nitric oxide and pro-inflammatory mediators while themselves being activated by the same signals [26,46,81]. Mechanistically, the recurring downstream mechanisms included NF-κB-driven transcription, NLRP3 inflammasome activation, cytokine and chemokine release, complement activation, and NRF2-related stress-response pathways across cerebral, retinal and optic nerve tissues [82,83,84,85,86].

Within the visual system the same loop was localized repeatedly in glaucomatous and diabetic neurodegeneration, with dysfunctional mitochondria and glial inflammation positioned at its centre and exosomal cargo proposed as a vehicle for propagating redox-inflammatory signals between ocular cell populations [87,88,89,90]. Therapeutically oriented work refined the cellular target, distinguishing resident microglia from monocyte-derived macrophages as “dual immune armies” and showing that restraining microglial activation (for example by inhibiting the mechanosensitive channel TRPV4, which curbed microglial ferroptosis) attenuated glaucomatous inflammation [91,92,93,94]. Astrocytic antioxidant defences partially restrain this loop [68,95].

### 3.5. Domain 4. Oxidative Stress in Specialized Axonal Projections and Cranial Nerve Vulnerability

While the optic nerve represented the principal focus of investigation, several studies widened the concept of redox vulnerability to encompass other long-range, energy-intensive neuronal projections. Shared pathological hallmarks included mitochondrial dysfunction, defective oxidative phosphorylation, axonal transport impairment, calcium dyshomeostasis, and restricted regenerative potential, underscoring common mechanisms of neurodegenerative susceptibility across neural systems [45,96,97,98]. Evidence of distinct cranial- and spinal-nerve involvement in progressive supranuclear palsy further suggested that selective axonal susceptibility is not confined to the optic nerve but may reflect a broader vulnerability of long neural projections to bioenergetic and oxidative stress [17,99].

**Figure 2:**
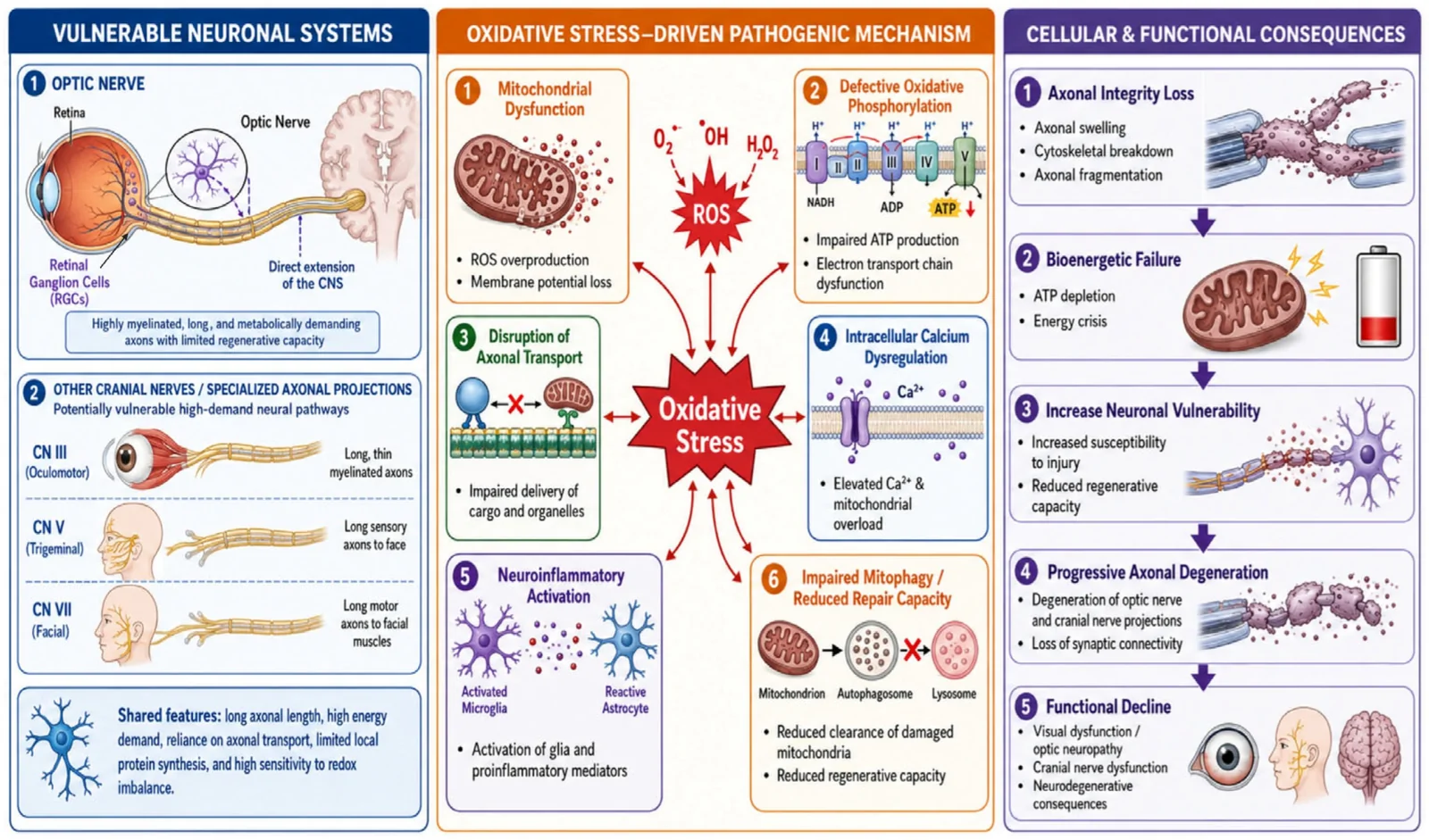
Oxidative stress in specialized axonal projections and cranial nerve vulnerability. Oxidative stress and redox imbalance contribute to neurodegeneration through interconnected pathological processes, including mitochondrial dysfunction, impaired oxidative phosphorylation, axonal transport disruption, calcium dyshomeostasis, neuroinflammatory activation, defective mitophagy and diminished regenerative capacity. The optic nerve represents the most extensively characterized model of this vulnerability, owing to the exceptional metabolic demands of RGC retinal ganglion cell axons, their dependence on mitochondrial trafficking and oxidative phosphorylation, and the unique accessibility of the visual system to non-invasive structural and functional assessment. Emerging evidence suggests that analogous bioenergetic and redox-dependent mechanisms may also underlie dysfunction in other cranial nerves and long-range neuronal projections, supporting the concept of a common framework of neurodegenerative susceptibility, although direct evidence beyond the visual system remains comparatively limited. Blue, orange, and purple panels represent vulnerable neuronal systems, pathogenic mechanisms, and cellular/functional consequences, respectively. Red arrows indicate reciprocal interactions, whereas purple arrows indicate sequential progression. *(Created in BioRender. TRAN VAN, Y. (2026) https://BioRender.com/tlcxcc7)*.

ALS offered a particularly illustrative example of the multisystem nature of neurodegeneration, as retinal and ocular abnormalities were documented in a disease historically considered a purely motor disorder. The observation of altered superoxide dismutase activity in tear fluid and peripheral blood further suggested that ocular biomarkers may provide a non-invasive window into systemic oxidative imbalance [100,101]. All these observations supported the review’s framing of redox imbalance as a pan-axonal pathogenic pathway, with the optic nerve serving less as an exception than as the best-instrumented example [25,46,73]. A conceptual synthesis of the redox-dependent mechanisms implicated in specialized axonal projection vulnerability is presented in Figure 2.

### 3.6. Domain 5. Optic Nerve and Retinal Neurodegeneration

As previously noted, the optic nerve and retina constituted the most densely represented anatomical domain among the included studies, while glaucoma emerged as the most frequently investigated condition. The literature consolidated a view of glaucoma as a multifactorial neurodegeneration (progressive RGC death, optic-nerve-head remodelling, glial activation, vascular dysregulation and chronic oxidative injury) rather than a disorder of intraocular pressure alone [19,26,102,103,104,105,106]. Enzymatic ROS sources were resolved in detail, with NADPH oxidase, and NOX4 in particular, identified as a principal generator of glaucomatous ROS whose pharmacological inhibition suppressed redox-sensitive transcription and attenuated ocular-hypertensive retinal injury [107,108]. This reframing is not new, and its foundational statements fall outside the window charted here [43,44]. Mechanistic studies increasingly clarified how oxidative injury contributes to RGCs death. Beyond classical apoptosis, oxidative stress was associated with regulated non-apoptotic cell-death pathways, including ferroptosis, pyroptosis and Drp1-related PANoptotic signalling, which are compared directly in Section Comparative Analysis of the Regulated Cell-Death Pathways [50,109,110,111,112,113,114,115,116,117]. In parallel, endogenous defences were mapped: pressure elevation triggered early activation of the NRF2-KEAP1-ARE antioxidant programme as a protective response, and normal-tension glaucoma, in which pressure is unremarkable, was used to argue that oxidative stress can act as a primary rather than secondary driver [118,119]. Additionally, Mendelian-randomization analysis provided human genetic evidence consistent with a causal contribution of oxidative stress to glaucoma risk. Glial compartments shared the vulnerability, with optic-nerve-head astrocytes protected from oxidative death by interventions that prevented caspase-3 activation and tau dephosphorylation [61,120]. Representative mechanisms are summarized in Table 2.

Beyond glaucoma, oxidative stress was implicated across a broader optic-neuropathy spectrum, including hereditary mitochondrial, ischemic, inflammatory, traumatic, and toxic/nutritional forms. Across these conditions, mitochondrial dysfunction recurred as a convergent lesion linking redox imbalance to RGCs vulnerability [21,25,58]. Experimental evidence further suggested that NRF2 activation, including with bardoxolone methyl and omaveloxolone, may improve RGCs survival in ischemic optic neuropathy models, while toxic insults such as organophosphate exposure also appeared to injure the optic nerve through oxidative pathways [125,126,127].

Diabetic retinal disease was reframed throughout the studies as a neurodegeneration that precedes overt microvascular change [29,128]. Hyperglycemia-driven ROS converged on ganglion-cell death by several routes: combined endoplasmic-reticulum and oxidative stress, thioredoxin-interacting-protein (TXNIP) signalling, β-catenin/GSK3β and ERK-Akt-mTOR dysregulation, and Nrf2/HO-1-regulated ferroptosis, the last suppressible by dopamine [128,129,130,131,132,133,134,135,136,137,138].

In the outer retina, the retinal pigment epithelium emerged as an oxidative battleground in age-related macular degeneration (AMD), through blood-retinal-barrier breakdown, DJ-1-dependent defences and ageing and iron-dependent lipid peroxidation driving ferroptosis [121,139,140,141,142,143]. Ferroptosis itself, iron-catalyzed phospholipid peroxidation, was among the fastest-growing threads, spanning diabetic and inherited retinal degenerations and bridging redox chemistry to a defined cell-death modality [121,123]. Models of photoreceptor and rod-cone degeneration reinforced the theme: gene therapy with oxidative-stress-resistance-1 (OXR1) retarded degeneration in the RD1 mouse while graded environmental light or high-fat feeding accelerated oxidative, inflammatory retinal neurodegeneration, and receptor-level interventions (e.g., galanin receptor 3 inhibition) slowed it [144,145,146]. Additionally, autophagy was repeatedly cast as the counter-regulatory arm [119,132].

Two overarching concepts emerged from this body of evidence. First, the exceptionally high metabolic demands of RGCs, which rely heavily on oxidative phosphorylation to sustain axonal transport, neurotransmission, and cellular homeostasis, render these neurons particularly susceptible to even subtle disturbances in bioenergetic balance. This intrinsic vulnerability is further exacerbated by glutamate-mediated excitotoxicity, creating a permissive environment for progressive neuronal injury and degeneration [22,23,147,148,149,150]. Second, the remarkable convergence of molecular and cellular pathways identified in optic neuropathies with those implicated in AD, PD and ALS supports the view that the optic nerve represents more than a simple analogue of CNS pathology. Rather, it has increasingly been proposed as a readily accessible and clinically relevant model for investigating the mechanisms, biomarkers, and progression of neurodegeneration within the broader CNS [25,71,151].

#### Comparative Analysis of the Regulated Cell-Death Pathways

Regulated cell death recurred across different domains 2, 3 and 5, but the mapped studies almost always discussed one pathway at a time, so the molecular features that actually distinguish them were rarely set against one another. All the pathways are compared directly (Table 3) rather than being described serially within each domain.

Three points follow from the comparison. First, the pathways differ fundamentally in where oxidation acts. In apoptosis, pyroptosis and necroptosis, ROS are upstream signals that licence a proteolytic or pore-forming execution step; the cell is not killed by oxidation. Ferroptosis is the exception: the peroxidized phospholipid is itself the lethal species [152], which is why it is the only pathway for which “antioxidant therapy” and “cell-death inhibition” are the same intervention. This distinction, rather than the shared vocabulary of ROS, is what determines whether a redox-directed drug can be expected to work. Second, the pathways are not mutually exclusive and the evidence that they are separable in vivo is weak. PANoptosis is defined by their convergence and most of the ocular evidence for ferroptosis and pyroptosis rests on pharmacological inhibitors (ferrostatin-1, necrostatin-1, MCC950) whose selectivity at the concentrations used in retinal preparations has not been established; the marker sets used (GPX4 depletion, ACSL4 induction, lipid-ROS probes) are also not individually specific. The apparent proliferation of distinct death modalities in the retina may therefore partly reflect reagent behaviour rather than biology. Third, the level of evidence is very irregular: ferroptosis is supported by convergent data across glaucoma, diabetic retinopathy and AMD, whereas PANoptosis in the retina rests, within this corpus, on a single mechanistic study. The therapeutic implication of the comparison is narrower than the enthusiasm of the primary literature suggests: only the ferroptotic arm currently offers a target whose modulation is both mechanistically coherent and pharmacologically feasible, and even there no ocular clinical data exist.

### 3.7. Domain 6. Oxidative Biomarkers

Biomarker discovery was among the most active areas mapped. The studies converged on a recognizable panel of oxidative readouts: 8-hydroxy-2′-deoxyguanosine (8-OHdG) and 8-hydroxyguanosine (8-OHG) for nucleic-acid oxidation; malondialdehyde (MDA), 4-hydroxynonenal (4-HNE) and F2-isoprostanes for lipid peroxidation; protein carbonyls; and glutathione- and superoxide-dismutase-based indices of antioxidant capacity [13,58,153,154,155,156,157,158]. These were assayed across an unusually wide range of matrices (serum, plasma, aqueous and vitreous humour, tear fluid, and cerebrospinal fluid) [101,159,160]. The main oxidative-stress biomarkers mapped in this review are summarized in Table 4, and the biological matrices in which they are measured are compared in Table 5.

Disease-specific applications were consistently reported across the literature, including serum oxidative and oxidative-inflammatory biomarkers in ocular hypertension and glaucoma, vitreous proteomic signatures in vitreoretinal disorders, and tear-fluid superoxide dismutase as a potential marker of systemic redox dysregulation in ALS [101,160,161,162]. Increasingly, oxidative biomarkers were integrated with imaging findings in age-related macular degeneration and explored as regulatory biomarkers, including specific microRNA species in primary open-angle glaucoma [159,165,171].

The comparison in Table 5 makes explicit a trade-off that the mapped literature acknowledges only implicitly: proximity to the target tissue and accessibility are inversely related across the whole matrix series. The matrices that can be sampled repeatedly and non-invasively (tear fluid, blood) are those furthest from the retina and the least organ-specific, whereas the matrices that report most directly on retinal redox state (vitreous, retinal tissue) are obtainable only from surgical or post-mortem material, that is, from precisely the patients in whom early detection is no longer the question. Aqueous humour and CSF occupy the middle ground at the cost of invasiveness and severe spectrum bias. No included study sampled more than two matrices concurrently in the same participants, so the assumption behind the minimally invasive strategy, that tear or serum redox markers track retinal redox state, is at present an inference rather than a finding.

Three analytical limitations constrain interpretation of this evidence. First, no oxidative-stress biomarker has a validated clinical decision threshold, and reported concentrations show substantial overlap between groups without independent diagnostic validation. Second, severe assay heterogeneity prevents reliable comparison or meta-analytic pooling, because the same analytes are measured using non-equivalent methods, kits, and reference standards. Third, systematic matrix interference and pre-analytical factors, including haemolysis, lipaemia, storage conditions, processing delays, and freeze–thaw cycles, may alter results more than the reported biological differences. Consequently, a substantial proportion of between-study variability is likely analytical rather than biological, making assay harmonization, reference materials, and external quality assessment essential prerequisites for clinical translation. A recurring challenge was the lack of standardization. Substantial heterogeneity in analyte selection, assay platforms, biological matrices, and reporting methods limited comparability across studies. Consequently, the field has progressively shifted toward multimarker approaches combining oxidative, inflammatory, mitochondrial, and neurodegenerative indicators rather than relying on single analytes [46,172]. While oxidative biomarkers show considerable diagnostic and prognostic potential, their routine clinical implementation remains contingent upon further analytical validation, methodological harmonization, and the establishment of standardized reference ranges.

### 3.8. Domain 7. Retina as a Biomarker of CNS Disease

A distinct and rapidly expanding strand reframed the retina not as a target of neurodegeneration but as a reporter of it. The argument rested on shared embryology, laminar architecture, neurovascular organization and molecular apparatus between retina and brain, which together make retinal change a plausible proxy for cerebral pathology accessible without biopsy or contrast administration [18,19,47,169].

The instrument of this paradigm was imaging. Optical coherence tomography and its angiographic extension (OCT/OCTA), retinal-nerve-fibre-layer and ganglion-cell-complex thickness, and retinal microvascular metrics were repeatedly associated with AD, PD, MS, frontotemporal degeneration and ALS [100,166,169]. Several reports indicated that retinal thinning and microvascular rarefaction may parallel, or even precede, detectable cerebral change and the “retina as a window to the brain” became an explicit organizing metaphor, extended even to glaucoma as a brain disease and to diabetic retinopathy as a source of neurodegenerative biomarkers [31,46,169]. Evidence that periodontitis-associated metabolic syndrome was linked to measurable retinal neurodegeneration further suggested that systemic redox-inflammatory burden can be reflected in retinal structure [46,173]. The concept has a longer history than the charted window: optical coherence tomography was proposed as a window onto the mechanisms of multiple sclerosis more than a decade before it [174]. Experimental work published outside the review period is also directly relevant to two of the mechanistic claims made here. In a lipopolysaccharide model, retinal function and recognition memory deteriorate in parallel and both are modulated by the same anti-inflammatory intervention within the same animals, which is the concordance design that the clinical literature has not yet supplied [175]. In traumatic injury, oxidative responses of the brain and the retina have been compared directly and the comparison shows both the shared redox chemistry that motivates the continuum and the tissue-specific differences that qualify it [72].

**Figure 3:**
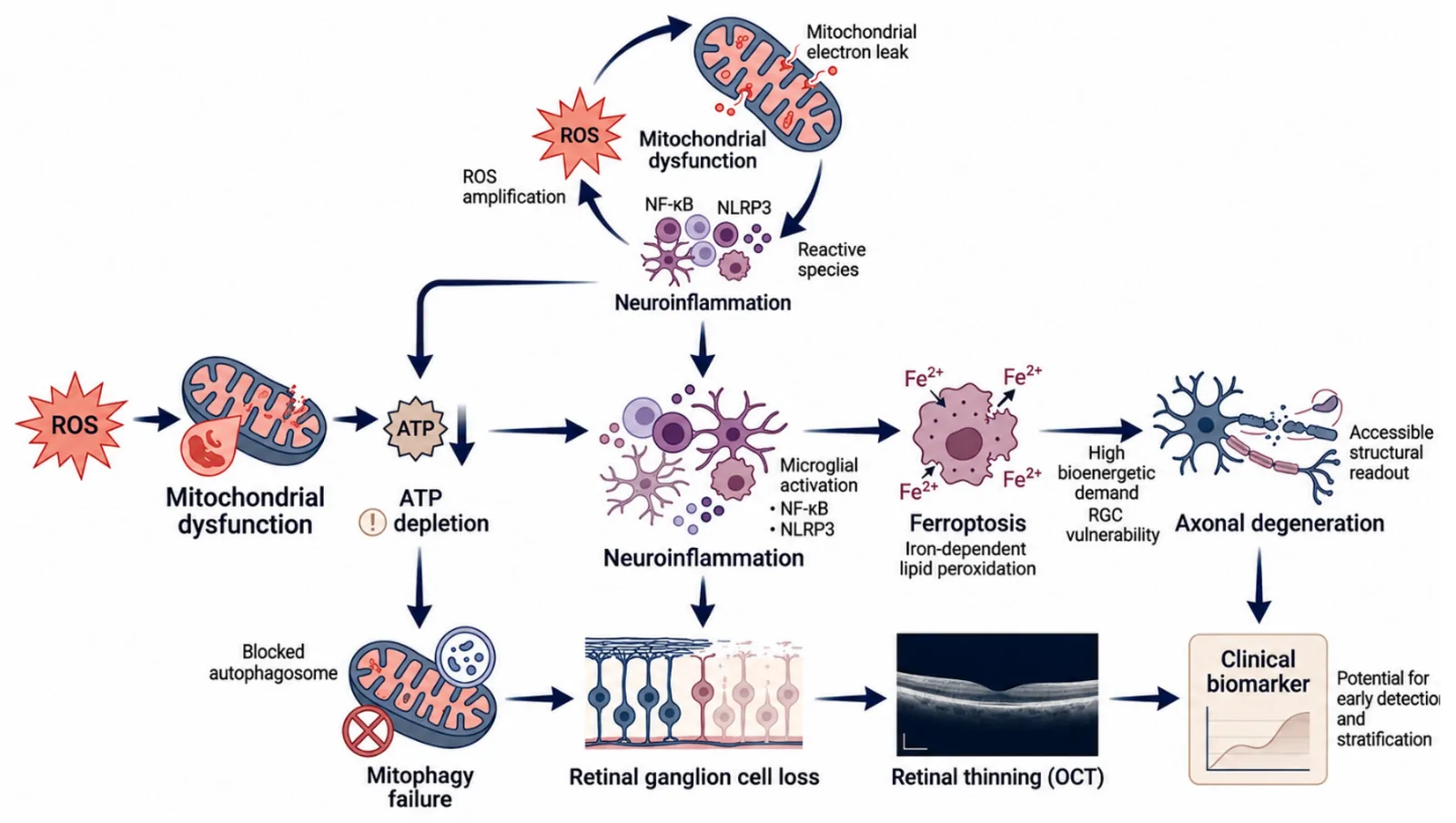
Conceptual framework of the oxidative stress-driven cascade linking mitochondrial dysfunction to retinal neurodegeneration and clinically detectable retinal biomarkers. Reactive oxygen species promote mitochondrial dysfunction, ATP depletion, mitophagy failure, neuroinflammation, ferroptosis, axonal degeneration, and retinal ganglion cell loss, ultimately leading to OCT-detectable retinal thinning as a potential clinical biomarker. Arrows indicate proposed directional relationships, whereas circular arrows denote a self-amplifying feedback loop among mitochondrial dysfunction, ROS, and neuroinflammation. Colors are used for visual differentiation only. *(Created in BioRender. TRAN VAN, Y. (2026) https://BioRender.com/ga9ezpo)*.

The literature consistently acknowledges important limitations, including the predominance of cross-sectional studies, heterogeneous imaging protocols and limited biomarker specificity. Nevertheless, the integration of artificial intelligence (AI) into ocular imaging and multimodal biomarker analysis represents a promising avenue for accelerating the clinical translation of retinal biomarkers. Recent advances in machine learning and deep learning have enabled increasingly accurate automated interpretation of optical coherence tomography (OCT), OCT angiography (OCTA), and fundus images, facilitating the detection of subtle structural and microvascular alterations that may escape conventional assessment. Moreover, AI algorithms are increasingly capable of integrating retinal imaging with fluid biomarkers, genetic data, clinical variables and other omics datasets to generate multidimensional biomarker signatures supporting patient phenotyping, endotyping, prognostic prediction, and therapeutic stratification. These multimodal analytical frameworks have the potential to advance precision medicine by improving individualized risk assessment, disease monitoring, and the identification of patients most likely to benefit from targeted neuroprotective and redox-modulating therapies. However, their routine clinical implementation will require robust external validation, standardized imaging protocols, transparent algorithm development and appropriate ethical and regulatory oversight. Consequently, retinal biomarkers should currently be regarded as complementary to established cerebral biomarkers within integrated multimodal diagnostic strategies [166,176,177]. The proposed oxidative stress-driven cascade linking mitochondrial dysfunction, neuroinflammation, RGC degeneration, and OCT-detectable retinal thinning is summarized in Figure 3.

#### Ethical, Regulatory and Standardization Considerations for AI-Based Retinal Biomarkers

Although autonomous retinal AI is already authorized for diabetic retinopathy screening, extending this model to prediction of cerebral neurodegeneration presents fundamentally different regulatory, technical, and ethical challenges. Unlike diagnosing existing retinal disease against an ophthalmic reference standard, predicting future neurodegeneration from retinal images is a prognostic claim concerning another organ and requires long-term prospective validation that is currently lacking. No included study externally validated a retinal algorithm against subsequent clinical neurodegenerative outcomes.

Translation is further limited by poor interoperability between proprietary imaging platforms, under-representation of diverse ancestry groups in normative databases, limited cross-device validation, and the absence of predefined protocols for model updating. These limitations create risks of reduced generalizability and systematic inequity. Ethical concerns are equally substantial because such algorithms could generate an unexpected dementia-risk prediction in asymptomatic individuals, raising issues related to informed consent, the right not to know, psychological harm, discrimination, and the biometric identifiability of retinal images. Explainability tools do not resolve the underlying uncertainty of an individual prediction.

Accordingly, retinal AI biomarkers should currently be regarded as research tools or as methods for enriching clinical-trial recruitment rather than as population screening instruments. Wider clinical use will require prospective longitudinal validation, demonstrated transportability across devices and populations, prespecified intended use, and a robust governance framework established before implementation.

### 3.9. Domain 8. Therapeutic and Translational Implications

Therapeutic and translational studies formed the final and one of the most heterogeneous domains, with interventions clustering into recognizable mechanistic families. Mitochondria-directed strategies included coenzyme Q10, shown to protect porcine retina from peroxide injury and, formulated as a vitamin-E-TPGS-conjugated eyedrop, to relieve neurodegeneration and mitochondrial dysfunction in the diabetic retina, alongside NAD^+^-repleting approaches in glaucoma [75,178,179,180]. Activators of the NRF2-ARE axis constituted a second family: bardoxolone methyl and omaveloxolone in ischemic optic neuropathy, pressure-validated NRF2 induction in ocular hypertension, nature-inspired activators protecting retinal explants, and the cysteine pro-drug N-acetyl-L-cysteine ethyl ester (NACET), which induced NRF2 and prevented retinal ageing and diabetic retinopathy [51,53,118,181,182].

Further families included free-radical scavengers and nitroxides (hybrid TEMPOL derivatives advanced specifically for ocular neurodegeneration), molecular hydrogen, manganese-porphyrin redox catalysts, and dietary polyphenols and nutraceuticals targeting the mitochondrion, of which resveratrol is a prototype [42,47,73,183,184,185,186]. Ferroptosis modulation, anti-inflammatory and microglia-directed agents, regenerative secretome-based therapy from mesenchymal stem cells, plasma-rich-in-growth-factors eyedrops, incretin- and dopamine-based metabolic agents, and OXR1 gene therapy rounded out the experimental armamentarium [66,109,122,187,188].

Across glaucoma, hereditary optic neuropathy, retinal degeneration and the cerebral proteinopathies, preclinical reports were strikingly concordant: lowering oxidative load preserved mitochondrial function, dampened neuroinflammation and improved neuronal survival [26,46,59]. Yet the same literature was uniformly sober about translation. Broad-spectrum antioxidant supplementation had largely disappointed in the clinic and reviews attributed the gap to biological heterogeneity, late intervention, incomplete biomarker validation, and the reality that oxidative stress is but one interlocking node of a network also comprising inflammation, mitochondrial failure, vascular compromise and proteostatic collapse [172]. The emerging prescription was precision, matching mechanism-specific redox, mitochondrial and imaging phenotypes to targeted interventions and to the disease stage at which they can still act [47,67].

Because the blood–retinal and blood–brain barriers exclude most of the molecules listed above, and because the choice between systemic, topical and intravitreal administration determines both the achievable tissue concentration and the trial design that can test it, the principal agents are compared on these grounds in Table 6.

The therapeutic literature separates into three clinically distinct groups. First, agents such as coenzyme Q10, resveratrol, other polyphenols and manganese porphyrins have generally failed to achieve adequate retinal exposure, so negative or absent clinical findings cannot be interpreted as evidence against the redox hypothesis. Second, therapies with adequate target delivery, including idebenone in Leber hereditary optic neuropathy and nicotinamide in glaucoma, have produced only modest effects on surrogate outcomes and therefore provide the most informative, although sobering, test of the hypothesis. Third, local approaches, including topical formulations, intravitreal ferroptosis inhibitors, secretome-based therapies and gene therapy, bypass systemic delivery barriers but remain supported mainly by preclinical evidence.

Local ocular administration can achieve substantially higher retinal drug concentrations while reducing systemic toxicity, but topical penetration to the posterior segment remains limited and formulation-dependent, whereas intravitreal injection carries procedural risks that may be difficult to justify for preventive treatment. Moreover, local ocular therapy does not address cerebral disease. Ophthalmology is therefore an appropriate setting in which to test redox-modulating neuroprotection because the target tissue is accessible and drug exposure and outcomes can be measured directly. However, a successful ocular intervention would not establish that the same target is pharmacologically tractable in the brain: the brain–retina continuum is mechanistic, not pharmacokinetic.

### 3.10. Consistency of the Evidence, and the Null and Contradictory Findings

The mapped literature presents a highly consistent association between oxidative stress and neurodegeneration, but this apparent convergence requires cautious interpretation. None of the 175 included records treated oxidative stress as epiphenomenal, reported harm from reducing oxidative load, or described a clearly ineffective preclinical redox-directed intervention. Such near-unanimity, particularly in a corpus dominated by reviews rather than independent clinical studies, raises concern about publication and citation bias. This concern is reinforced by the clinical trial record, which has been predominantly negative: α-tocopherol failed to slow early Parkinson disease progression, high-dose coenzyme Q10 was stopped for futility, MitoQ showed no benefit, idebenone missed its primary endpoint in Leber hereditary optic neuropathy, and AREDS2 found no additional benefit from lutein, zeaxanthin, or omega-3 supplementation [189,190,193,194,195,196]. Moreover, meta-analytic evidence has shown no mortality benefit from antioxidant supplementation and possible harm with β-carotene, vitamin E, and high-dose vitamin A, emphasizing that physiological reactive oxygen species signalling cannot be suppressed without potential consequences [197].

The biomarker literature is similarly heterogeneous. Oxidative markers such as superoxide dismutase have been reported as both increased and decreased, while retinal structural studies in Alzheimer disease and mild cognitive impairment show statistically significant pooled changes but substantial heterogeneity and marked overlap between cases and controls, limiting individual-level diagnostic usefulness [198,199]. Most human evidence is cross-sectional and cannot distinguish whether oxidative stress is causal or a consequence of tissue injury. Mendelian randomization evidence in glaucoma and inherited mitochondrial optic neuropathies provides some causal support, but these findings are condition-specific and cannot be generalized to common neurodegenerative disorders. Alternative pathogenic mechanisms, including protein aggregation, neuroinflammation, lysosomal and proteostatic failure, vascular dysfunction, glymphatic impairment, and genetic susceptibility, are at least as strongly supported in the wider literature [200,201]. The most defensible conclusion is therefore that oxidative stress is a reproducible, biologically coherent, and potentially modifiable component of neurodegeneration, but its independent causal contribution to common human diseases remains uncertain and may be overstated by methodological and publication-related convergence.

## 4. Discussion

This scoping review mapped a large, methodologically heterogeneous body of contemporary evidence (2020–2026) onto eight pre-specified domains and, in doing so, disclosed a field that has organized itself around a single proposition, that oxidative stress is a convergent, causally upstream mechanism of neurodegeneration which operates with particular force in the visual system, while subjecting that proposition to remarkably little adversarial testing. Three observations recurred across the corpus, although recurrence in a literature of this composition is weaker evidence than it appears. First, oxidative injury, mitochondrial dysfunction and neuroinflammation were reported together far more often than separately. Second, the RGC and optic nerve recurred as the compartment in which these mechanisms are most measurable and most manipulable. Third, the same molecular vocabulary including NRF2, NAD^+^, Drp1, NLRP3, and ferroptosis, appeared whether the disease under study was cerebral or ocular, which has been read as supporting a brain-retina continuum rather than a dichotomy. This last observation is the most equivocal of the three. A shared vocabulary may indicate a shared mechanism, or it may indicate a shared set of assays, antibodies and inhibitors applied to two tissues by investigators reading the same reviews. Distinguishing the two requires evidence that brain and retina respond concordantly within the same individuals, which the mapped literature does not provide.

The most important conceptual yield of the mapping was the consolidation of an oxidative stress-mitochondria-neuroinflammation triad contributing to the pathology of various neurodegenerative diseases. The directionality reported across studies was circular rather than linear: mitochondrial electron leak generates ROS [62,63]; ROS damage mitochondrial lipids, proteins and DNA, further impairing respiration [62,202]; and oxidatively stressed neurons and glia activate NF-κB- and NLRP3-dependent inflammatory programmes that themselves produce additional reactive species [69,88]. Section 3.3 and Section 3.4 document this self-potentiation in cerebral and ocular disease alike [11,69,87,88,90]. The therapeutic significance of a circular pathology is considerable. Any single-node intervention is liable to be buffered by the others, an argument that may explain the modest clinical returns of isolated antioxidant supplementation and that motivates combination or upstream strategies such as NRF2 restoration and mitochondrial stabilization [78,172].

A second synthesis concerns why the optic nerve recurred so insistently. This configuration converts the optic nerve into a biological amplifier of bioenergetic stress and, because it is transparent to light and surgically accessible, into an unusually well-instrumented one. The inherited mitochondrial optic neuropathies provide proof of principle that isolated respiratory-chain dysfunction is sufficient to kill RGCs [22], while the co-occurrence of ALS with LHON and the broader retinal involvement of ALS indicate that the optic nerve also reports systemic axonopathy [54,100,151]. The implication is that the optic nerve should be read not as an ophthalmological special case but as a tractable window onto mechanisms otherwise observable only at autopsy in the brain.

A notable temporal trend was the ascent of regulated cell death, particularly ferroptosis, as an organizing concept. Where earlier accounts of oxidative neuronal death defaulted to apoptosis, the mapped literature increasingly invoked iron-dependent phospholipid peroxidation and hybrid death programmes (Drp1-linked PANoptosis, glaucomatous ferroptosis and pyroptosis, iron-mediated peroxidation in the ageing macula and ferroptotic cascades in diabetic retinopathy) [50,121,122,123]. This is a genuine mechanistic refinement, and ferroptosis is in principle tractable through iron chelation, glutathione-peroxidase-4 support and lipophilic radical trapping. The unresolved question is not whether these pathways are engaged but whether they are separable in vivo: the ocular evidence rests largely on inhibitors of uncertain selectivity, so tractability is at present a claim about tool compounds rather than about drugs [92,191]. Its association with microglial ferroptosis further links oxidative injury to neuroinflammatory amplification in glaucoma, suggesting that the modality of cell death, not only the magnitude of redox burden, may become a meaningful therapeutic variable [92].

The biomarker and “retina-as-window” domains outline a promising pathway toward clinical translation while highlighting its major challenge. Current evidence indicates that oxidative injury generates measurable molecular signatures in accessible biological matrices and structural alterations detectable by OCT/OCTA [13,171,173]. However, neither fluid nor imaging biomarkers have achieved sufficient standardization for individual-level clinical decision-making because of methodological heterogeneity, predominantly cross-sectional designs, and limited specificity [153,167,172]. The findings suggest that no single biomarker is likely to be adequate. Instead, multimodal approaches integrating oxidative, inflammatory, mitochondrial, and neurofilament biomarkers with retinal structural and vascular imaging may offer greater diagnostic and prognostic value [47,129,160]. Longitudinal studies will be essential to determine whether retinal redox alterations precede, accompany, or follow cerebral neurodegeneration.

### 4.1. Retinal Biomarkers Versus Established CNS Biomarkers: A Critical Comparison

Established CSF, plasma, and PET biomarkers of neurodegeneration now benefit from analytical standardization, external quality assessment, strong individual-level discrimination, and prospective clinical validation. By contrast, no oxidative or retinal marker reviewed here meets comparable standards. Although meta-analyses show significant thinning of the retinal nerve fibre layer and ganglion-cell complex in Alzheimer disease and mild cognitive impairment, the effect sizes are small, heterogeneity is high, and diagnostic performance is generally insufficient for individual classification [198,199]. Specificity is further limited because retinal thinning also occurs with glaucoma, diabetes, hypertension, ischaemia, myopia, and ageing, while measurements are not fully transferable across imaging devices. Most importantly, no included study prospectively linked retinal findings to adjudicated cerebral outcomes or directly compared retinal biomarkers with established plasma markers in the same participants. Retinal imaging nevertheless offers unique advantages as a non-invasive, inexpensive, repeatable, and widely accessible measure of central neural tissue. Its most defensible current roles are therefore as a triage or trial-enrichment tool and as a longitudinal structural endpoint, rather than as a stand-alone diagnostic or population screening test. Retinal and oxidative biomarkers should be regarded as complementary to established CNS biomarkers, while the eye-as-window hypothesis remains to be prospectively demonstrated.

The therapeutic literature supports a nuanced interpretation. Although reducing oxidative stress consistently confers neuroprotection in preclinical models, broad-spectrum antioxidant strategies have largely failed to translate into clinical benefit [1,68,153,203]. Three factors may explain this discrepancy. First, mechanism-specific interventions, including NRF2 activators, NACET, coenzyme Q10, NAD^+^ restoration, and mitochondrial biogenesis enhancers, appear better suited to interrupt self-perpetuating redox dysfunction than conventional radical scavengers [22,30,78]. Second, timing is critical, as oxidative injury likely exerts its greatest influence before irreversible neuronal and axonal loss, emphasizing the importance of early detection [25,72,153]. Third, patient stratification is essential, given the marked biological heterogeneity of neurodegenerative disorders.

These considerations also support a broader transition from empirical antioxidant supplementation toward precision neurodegeneration medicine. Rather than treating oxidative stress as a universal therapeutic target, future interventions should be guided by careful patient phenotyping and biological endotyping, integrating molecular, metabolic, imaging, and fluid biomarkers to identify individuals in whom oxidative stress represents a dominant pathogenic mechanism. Biomarker-guided therapeutic selection and longitudinal patient stratification may enable the identification of disease-specific redox signatures, optimize treatment timing, and facilitate personalized multimodal interventions targeting the complex interplay among oxidative stress, mitochondrial dysfunction, neuroinflammation, and regulated cell-death pathways. Such precision-based approaches are increasingly regarded as essential for improving the clinical translation of redox-modulating therapies. Redox-targeted therapies are most likely to benefit phenotypes characterized by elevated oxidative burden, mitochondrial dysfunction, or specific cell-death pathways [13,129]. Notably, emerging molecular targets (NOX/NOX4, Drp1, TXNIP, GPX4/ferroptosis, and complement pathways) and innovative ocular delivery systems, including topical and sustained-release formulations, support the feasibility of precision redox-based therapies, particularly in ophthalmology [47,107,204].

### 4.2. Why Antioxidant Strategies Have Not Translated

The reviews mapped here describe the clinical antioxidant record as “disappointing” and attribute it to heterogeneity, late intervention and incomplete biomarker validation. That account is incomplete, and it is also self-serving, because every explanation it offers leaves the hypothesis intact. Five explanations are compatible with the evidence, and they carry very different consequences.

First, the drug never arrived. For coenzyme Q10, the polyphenols and the metalloporphyrins, systemic administration cannot deliver a meaningful concentration to the retina or brain. Second, the intervention arrived too late. Oxidative injury is upstream and cumulative, so a trial that begins after symptomatic diagnosis tests salvage, not prevention; this is a real limitation, but it is also unfalsifiable in its usual form, and it will remain so until a trial enriched for preclinical disease is actually run. Third, the population was wrong. Redox-targeted therapy should benefit the subset with a demonstrable oxidative phenotype, yet not one of the trials in this literature stratified on a baseline redox measure, which is unsurprising, since no validated redox phenotype exists. The stratification argument therefore presupposes the biomarker programme that has itself not succeeded. Fourth, the model was wrong. Efficacy is routinely established by a short intervention in a young rodent subjected to an acute, supraphysiological insult (microbead-induced ocular hypertension, high-dose LPS, streptozotocin) and then extrapolated to a decades-long, low-grade process in a seventy-five-year-old with comorbidity and polypharmacy. Concordance between these two would be surprising; discordance is not evidence of a failure of execution.

The fifth explanation is the one the corpus does not entertain: that less reactive oxygen is not, in general, better. ROS are signalling molecules as well as toxins. NRF2 induction is itself ROS-dependent, NOX-derived signalling maintains neural progenitor populations, and the adaptive response to mild redox stress is protective. Broad-spectrum scavenging removes the signal along with the damage, and the meta-analytic finding that β-carotene, vitamin E and high-dose vitamin A supplementation are associated with increased rather than decreased mortality is the predictable consequence, not an anomaly [197]. This reframes the therapeutic objective from suppression to restoration of redox homeostasis, and it explains why the interventions that retain promise are the ones that modulate endogenous defence (NRF2 activation, NAD^+^ repletion, GPX4 support) rather than those that titrate radicals directly. It also implies that the failure of the scavengers is not evidence against redox biology; it is evidence against a naive version of it that the field has largely abandoned in principle while continuing to cite in practice.

A sixth possibility should be stated plainly because the mapped literature never states it: oxidative stress may not be a dominant independent driver in the common neurodegenerative diseases. Nothing in the evidence assembled here excludes that reading, and the only disease-modifying therapies so far approved in this field target amyloid rather than redox. Discriminating among these six explanations does not require more preclinical work. It requires trials designed to fail informatively: with target engagement measured in the relevant compartment so that a null result distinguishes a wrong target from an undelivered drug, with stratification on a baseline redox phenotype rather than a clinical diagnosis, with pre-specified futility rules, and with publication of null results. LHON and glaucoma are the natural settings for such trials, not because the eye is special, but because they are the only places where the drug can be put where the target is and the effect measured directly.

### 4.3. Strengths and Methodological Considerations

Consistent with the methodological frameworks of Arksey and O’Malley, Levac et al., and the Joanna Briggs Institute, the objective was to map the scope and structure of the evidence rather than quantify effect sizes [33,34,35]. Accordingly, no meta-analysis or formal risk-of-bias assessment was performed, and the observed consistency reflects convergence across studies rather than pooled estimates. The review is strengthened by its comprehensive scope, its PCC-based and PRISMA-ScR-compliant methodology [36], and its integration of neurodegeneration and visual neuroscience, two closely related fields that are often investigated separately.

### 4.4. Limitations of This Scoping Review

No quality appraisal or risk-of-bias assessment was performed, no meta-analysis was undertaken and no effect sizes are reported. Given the analytical heterogeneity, non-interchangeable assays for the same analyte, absent reference materials, unreported coefficients of variation, pooling would not have been defensible. The consequence is that this review can describe the direction of the literature but cannot quantify the magnitude of any association and the reader should not infer effect size from the frequency with which a finding is reported.

Study designs were heterogeneous and review articles were charted alongside primary studies. Reviews formed the largest single category (69 of 175 records).

Inclusion was restricted to English-language publications. This is a more consequential restriction here than in many fields: a large share of the primary experimental work in glaucoma and retinal redox biology is conducted in China and Japan, and glaucoma was the most represented condition in this corpus. Relevant work published in Chinese, Japanese or Korean was systematically excluded, and the direction of any resulting bias cannot be estimated.

The 2020–2026 window keeps the map current but introduces a structural bias whose direction is knowable. Almost the entire negative clinical trial record in this field pre-dates 2020, whereas the positive preclinical literature is overwhelmingly recent. A window that admits the enthusiasm but excludes the disappointments will systematically over-represent the hypothesis. Publication bias cannot be formally assessed in a scoping review. Indirect evidence of it is nonetheless present in the corpus itself: no included record reported a null or contradictory preclinical result. Grey literature, trial registries, conference proceedings and non-indexed reports were not searched, so completed but unpublished negative studies are invisible to this map by construction.

The eight thematic domains were defined a priori but overlap substantially: mitochondrial dysfunction, NRF2 signalling, inflammation and ROS recur in domains 2, 3 and 5. Assignment of a study to a single domain required judgement, was not formally tested for inter-rater reliability, and the domain structure itself embodies the mechanistic framework the review sets out to examine, a circularity that a scoping review can disclose but not remove.

Finally, the evidence base is preclinical: of 175 records, 98 were animal or in vitro studies and 6 were clinical. Every statement about clinical utility in this review therefore rests on roughly 3% of the corpus, and the review was not prospectively registered, which affords less protection against post hoc modification of the domains than a registered protocol would.

### 4.5. Research Gaps and Future Directions

A major translational challenge highlighted by the available evidence is the persistent discrepancy between the robust neuroprotective effects of antioxidant therapies observed in experimental models and their generally modest clinical efficacy. Several factors likely contribute to this gap, including inadequate patient stratification, initiation of treatment after irreversible neuronal damage has occurred, limited penetration of therapeutic agents into the CNS and retinal tissues, and the reliance on single-target interventions to modulate a highly interconnected pathogenic network. Indeed, oxidative stress rarely acts in isolation but interacts dynamically with mitochondrial dysfunction, neuroinflammation, impaired proteostasis, vascular dysregulation, and multiple regulated cell-death pathways. These considerations suggest that future therapeutic strategies should move beyond nonspecific antioxidant supplementation toward mechanism-based, biomarker-guided, multimodal interventions administered at the earliest stages of neurodegeneration.

The evidence map highlights several priority areas for future research. Longitudinal studies correlating retinal redox and structural alterations with subsequent cerebral neurodegeneration are needed to validate the retina-as-window hypothesis. Biomarker development requires assay standardization and harmonized reference ranges to enable the validation of multimodal panels. Mechanistic investigations should focus on identifying the specific cell-death pathways and molecular targets modulated by redox-based interventions, as well as the optimal stage for therapeutic intervention. Finally, clinical translation will require biomarker-guided and stage-specific trials, particularly in glaucoma and hereditary optic neuropathies, where disease phenotypes are measurable, target tissues are accessible, and sustained intraocular drug delivery is already feasible [61,78,192].

## 5. Conclusions

This scoping review shows that the evidence linking oxidative stress to neurodegeneration is extensive but remains predominantly preclinical. Across 175 included records, animal and in vitro studies greatly outnumbered clinical investigations, while human evidence was concentrated mainly in glaucoma and diabetic retinopathy and was largely cross-sectional. Oxidative stress emerged as a biologically coherent component of a self-amplifying network involving mitochondrial dysfunction, neuroinflammation, and regulated cell-death pathways. However, current evidence does not establish it as the predominant causal driver of common neurodegenerative diseases. Direct causal support remains limited, clinical trials have generally been inconclusive or negative, and the scarcity of null findings raises concern about selective reporting.

The optic nerve and retina were the most intensively studied compartments and represent the most accessible sites for measuring and manipulating redox-related processes. Nevertheless, the retina cannot yet be considered a validated individual-level surrogate of cerebral neurodegeneration, because paired retinal and cerebral redox assessments, comparisons with established fluid biomarkers, validated thresholds, and adequate control of major confounders are lacking.

Future research should prioritize longitudinal studies evaluating retinal and cerebral changes concurrently, analytical harmonization and external quality assessment of biomarkers, trials that confirm target engagement in the relevant tissue, and patient stratification according to baseline redox phenotype rather than diagnosis alone. The eye remains a uniquely accessible part of the central nervous system and therefore a rational setting in which to test the oxidative hypothesis. The promise of the brain–retina continuum is substantial, but the decisive translational evidence has not yet been generated.

## Figures and Tables

**Table 1:** Characteristics of representative included studies. The table reports 18 selected articles representing different study designs, anatomical compartments, and mechanistic themes, including clinical, animal, in vitro, in silico, human genetic, and review studies.

Author (Year)	Country	Study Design	Disease/Model	Main Findings
Ahmad et al. (2024) [14]	Multinational (Europe)	Clinical (CSF cohort, MCI)	Alzheimer’s disease/MCI	Oxidative-stress and inflammatory metabolites associate with AD cerebrospinal-fluid biomarkers in MCI.
Cheng et al. (2022) [16]	USA	Narrative review	Neurodegeneration/axon	Axonal mitochondrial maintenance and bioenergetics determine neuronal vulnerability and regenerative capacity.
Calkins et al. (2021) [23]	USA	Narrative review	Glaucoma	Frames glaucoma as a progressive RGC neurodegeneration with adaptive and maladaptive responses to metabolic and oxidative stress along the optic projection.
Buonfiglio et al. (2023) [25]	Germany	Narrative review	Optic nerve diseases	Oxidative stress is a suitable therapeutic target across optic nerve diseases.
Tezel et al. (2022) [26]	USA	Narrative review	Glaucoma	Synthesizes molecular regulation of neuroinflammation (glia, complement, oxidative signalling) as a treatment target in glaucoma.
Banna et al. (2024) [32]	Australia	Narrative review	Eye-brain axis	Reviews retinal imaging as a non-invasive window to brain health.
Zeng et al. (2023) [50]	China	Experimental (in vivo + in vitro)	Glaucoma	Pathologically high IOP drives Drp1-dependent mitochondrial dysfunction and RGC PANoptosis; Drp1 inhibition is protective.
Realini et al. (2025) [51]	Italy	Animal (mouse) + in vitro	Retinal ageing/diabetic retinopathy	The cysteine pro-drug NACET induces NRF2 and prevents retinal ageing and diabetic retinopathy.
Rosenkranz et al. (2021) [52]	Germany	Animal (EAE mouse)	Multiple sclerosis	Enhancing neuronal mitochondrial activity protects against inflammatory neurodegeneration.
Chen et al. (2024) [53]	China	Narrative review	Glaucoma	Reviews ferroptosis and pyroptosis as regulated-necrosis pathways driving RGC death.
Amore et al. (2023) [54]	Italy	Clinical (case report)	ALS + LHON	Co-occurrence of ALS and LHON suggests mitochondrial dysfunction as a shared phenotypic modifier.
Surma et al. (2023) [55]	USA	In vitro (hPSC-derived RGCs)	RGC/optic neuropathy	Enhanced mitochondrial biogenesis promotes RGC neuroprotection against oxidative injury.
Kutsyr et al. (2020) [56]	Spain	Animal (mouse)	Retinal degeneration	Gradually increased environmental light induces oxidative stress/inflammation and accelerates retinal neurodegeneration.
Usategui-Martín et al. (2022) [57]	Spain	In vitro/ex vivo (organotypic retina)	Retinal neuroprotection	MSC secretome modulates oxidative stress, autophagy and programmed cell death to protect the retina.
Sanz-Morello et al. (2021) [58]	Denmark	Narrative review	Optic neuropathies	Redox imbalance contributes across the optic-neuropathy spectrum.
Catalani et al. (2023) [59]	Italy	Narrative review	RGC degeneration	Targeting mitochondrial dysfunction and oxidative stress to prevent RGC neurodegeneration.
Korczowska-Łącka et al. (2023) [60]	Poland	Clinical (patient biomarker study)	Neurological diseases	Selected oxidative-stress and energy-metabolism biomarkers are altered across neurological diseases.
Shi et al. (2024) [61]	China	Human genetic (Mendelian randomization)	Glaucoma	Genetic evidence supports a causal effect of oxidative stress on glaucoma risk.

**Table 2:** Core oxidative mechanisms in optic-nerve and retinal neurodegeneration.

Mechanism Domain	Key Mediators/Molecular Effectors	Role in Optic-Nerve and Retinal Neurodegeneration	Refs
ROS/RNS and redox imbalance	Superoxide (O_2_•^−^), H_2_O_2_, hydroxyl radical, peroxynitrite (ONOO^−^); NADPH oxidases (NOX1/2/4), xanthine oxidase, uncoupled NOS; lipid peroxidation, protein carbonylation, 8-OHdG; counter-regulated by the NRF2-KEAP1-ARE axis.	Macromolecular damage and RGC apoptosis; acts as both an initiator and a feed-forward amplifier of degeneration.	[3,10,67,108,118]
Mitochondrial dysfunction	Electron-transport-chain/OXPHOS deficits, membrane-potential collapse, Drp1-mediated fission, mtDNA damage, NAD^+^ depletion, impaired biogenesis.	Bioenergetic failure of high-demand RGCs; a major ROS source; drives apoptosis/PANoptosis and axonal degeneration.	[22,45,49,50,55]
Neuroinflammation	Microglia, astrocyte and Müller-glia activation; NF-κB, NLRP3 inflammasome, IL-1β/TNF-α; complement; galectin-3; exosomal signalling; TRPV4.	Establishes a self-reinforcing oxidative-inflammatory loop that amplifies RGC injury.	[7,9,26,82,83,88,90,92]
Ferroptosis	Iron overload, ACSL4/LPCAT3-dependent PUFA-phospholipid peroxidation, system xc^−^/GSH/GPX4 axis, FSP1-CoQ10 pathway.	Iron-dependent death of RGCs, RPE and photoreceptors in glaucoma, diabetic retinopathy and AMD.	[121,122,123]
Mitophagy/autophagy (quality control)	PINK1/Parkin, OPTN, BNIP3/NIX-mediated axonal mitophagy; autophagic flux.	Clears damaged mitochondria; failure causes ROS accumulation and glaucomatous degeneration.	[47,76,124]

**Table 3:** Comparative molecular features of the regulated cell-death pathways implicated in oxidative retinal and cerebral neurodegeneration.

Feature	Apoptosis	Ferroptosis	Pyroptosis	Necroptosis	PANoptosis
Defining lesion	Caspase-dependent, orderly dismantling of the cell.	Iron-catalyzed peroxidation of polyunsaturated-fatty-acid-containing membrane phospholipids.	Inflammasome-driven, gasdermin-mediated plasma-membrane pore formation.	RIPK3/MLKL-driven membrane rupture, engaged when caspase-8 is inhibited.	Simultaneous engagement of the pyroptotic, apoptotic and necroptotic machinery within a single PANoptosome.
Core executioners	BAX/BAK, cytochrome c, APAF1, caspase-9, caspase-3/7.	ACSL4, LPCAT3, lipoxygenases/POR; restrained by system xc^−^, GSH, GPX4 and the FSP1–CoQ10 and GCH1–BH4 axes.	NLRP3 or AIM2, ASC, caspase-1 (or caspase-4/5/11), gasdermin D.	RIPK1, RIPK3, MLKL.	ZBP1/RIPK1/RIPK3/caspase-8/NLRP3 PANoptosome; in the retina, Drp1-dependent mitochondrial fission is the reported upstream trigger.
Relationship to redox imbalance	ROS act upstream, mainly via mitochondrial outer-membrane permeabilisation. Oxidation is permissive, not executing.	Redox chemistry is the execution mechanism itself: lipid hydroperoxides are the lethal species. This is the only pathway in which oxidative stress is not merely an initiator.	ROS prime and activate NLRP3 but do not execute the cell. Oxidation is a licencing signal.	ROS stabilize and amplifies RIPK1/RIPK3 assembly; contributory rather than obligatory.	ROS act at several nodes at once; the prevailing redox and iron burden appears to determine which arm predominates.
Iron dependence	No.	Obligatory.	No.	No.	Not obligatory; iron availability may bias the programme towards its ferroptotic arm.
Membrane integrity and immunogenicity	Preserved until late; immunologically silent.	Lost; releases DAMPs and oxidized lipid species; immunogenic.	Lost early; releases mature IL-1β and IL-18; strongly inflammatory.	Lost; DAMP release; inflammatory.	Lost; combined DAMP and cytokine output; strongly inflammatory.
Principal evidence in the visual system	The historical default account of RGC loss in glaucoma and optic neuropathy.	RGC, RPE and photoreceptor death in glaucoma, diabetic retinopathy and AMD; microglial ferroptosis downstream of TRPV4.	NLRP3/caspase-1 activation in glaucomatous and diabetic retinal injury.	Reported mainly as a component of mixed death programmes; the least characterized of the five in this corpus.	A single mechanistic study: IOP-driven, Drp1-dependent RGC PANoptosis.
Pharmacological tractability	Caspase inhibition preserves cell counts but has not preserved function in vivo.	Iron chelation, GPX4 support and lipophilic radical trapping (ferrostatin-1, liproxstatin-1); the most tractable of the five.	NLRP3 inhibitors, IL-1 blockade, gasdermin-D inhibitors.	RIPK1/RIPK3 inhibitors (e.g., necrostatin-1); no ocular clinical data.	Only upstream targeting is available (Drp1 inhibition, ZBP1); no selective inhibitor and no clinical data.
Strength of the evidence mapped here	Extensive and well replicated, but largely descriptive.	Expanding rapidly; almost entirely preclinical and heavily dependent on pharmacological inhibitors of imperfect selectivity, so the pathway may be over-called.	Moderate; preclinical.	Limited.	Single-study; the concept has not yet been independently replicated in the visual system and should be regarded as provisional.

**Table 4:** Oxidative-stress biomarkers across the brain-retina neurodegenerative continuum. No validated clinical decision threshold (“critical value”) is listed for any analyte because none exists. The assignments in this column describe the use for which each analyte is currently being investigated, not a use for which it is validated; every entry other than retinal structural imaging should be read as research-grade.

Biomarker	Matrix	Disease/Context	Clinical Significance	Primary Intended Use	Ref.
8-OHdG/8-OHG (oxidative DNA/RNA damage)	Retina, serum, CSF	Glaucoma, AD, neurodegeneration	Marker of oxidative nucleic-acid damage; localizes to RGC mitochondria; candidate progression marker.	Diagnostic (exploratory); progression monitoring.	[14,49,60]
Malondialdehyde (MDA)	Serum, aqueous humour	Ocular hypertension, POAG	Lipid-peroxidation marker elevated in glaucoma/OHT; component of combined oxidative-stress panels.	Diagnostic adjunct; therapeutic monitoring.	[13,160,161]
4-Hydroxynonenal (4-HNE)	Retinal/vitreous tissue	Vitreoretinal & neurodegenerative disease	Reactive lipid-peroxidation aldehyde; indicator of tissue oxidative damage.	Mechanistic and tissue research only.	[6,162]
F2-isoprostanes	Plasma, CSF	Neurodegeneration	Reference lipid-peroxidation biomarker reflecting systemic oxidative load.	Reference analyte for systemic oxidative load; trial pharmacodynamics.	[13,60]
Protein carbonyls	Serum, CSF	MCI, neurological disease	Protein-oxidation marker; preclinical indicator of cognitive impairment.	Exploratory diagnostic (MCI); research use.	[60,153]
Glutathione (GSH/GSSG ratio)	Blood, aqueous humour	Glaucoma	Reflects antioxidant reserve; a reduced ratio indicates redox imbalance.	Prognostic; therapeutic monitoring of redox reserve.	[13,160]
Superoxide dismutase (SOD) activity	Tear fluid, blood	ALS, glaucoma	Antioxidant-enzyme activity; minimally invasive (tear) readout of systemic redox state.	Exploratory diagnostic; non-invasive monitoring.	[101,161]
NAD^+^/NADH redox state	Retina, blood	Glaucoma	Bioenergetic/redox biomarker and therapeutic target.	Target engagement/pharmacodynamic readout.	[77,163]
Thioredoxin-interacting protein (TXNIP)	Retinal tissue	Diabetic retinopathy	Links hyperglycemia to oxidative/inflammatory RGC injury; therapeutic target.	Preclinical target identification; tissue only.	[164]
MicroRNAs (redox-related miRs)	Aqueous humour, serum	POAG	Regulatory biomarkers of oxidative-stress signalling.	Exploratory diagnostic and stratification.	[165]
Vitreous oxidative/inflammatory proteome	Vitreous humour	Vitreoretinal disease	Proteomic signature integrating oxidative, inflammatory and neurodegenerative markers.	Discovery proteomics; intra-operative research.	[162]
Iron/lipid-peroxidation (ferroptosis) indices	Retina (tissue/imaging)	AMD, diabetic retinopathy	Indicate iron-dependent lipid peroxidation and ferroptotic burden.	Preclinical mechanistic; imaging indices exploratory.	[121,123]
Retinal structural imaging (RNFL, GCC, OCT/OCTA)	Retinal imaging (in vivo)	AD, PD, MS, glaucoma	Non-invasive structural/vascular biomarkers paralleling CNS neurodegeneration.	Diagnostic and progression monitoring; candidate trial endpoint.	[166,167,168,169,170]
Integrated multimarker panels	Serum/CSF + imaging	Neurodegeneration (general)	Combine oxidative, inflammatory, mitochondrial and neurofilament markers for staging and stratification.	Stratification and staging.	[13,32,153]

**Table 5:** Comparative characteristics of the biological matrices used for oxidative-biomarker measurement across the brain-retina continuum.

Matrix	Access and Invasiveness	Proximity to the Target Tissue	Principal Oxidative Analytes	Key Advantages	Key Limitations and Interfering Factors
Tear fluid	Non-invasive; Schirmer strip or capillary; freely repeatable.	Ocular surface only; no contact with retina or optic nerve.	SOD and other antioxidant enzymes, MDA, 4-HNE adducts, cytokines.	The only truly non-invasive fluid matrix; suited to repeated sampling and to screening; feasible in patients, such as those with ALS, in whom repeated venepuncture or lumbar puncture is burdensome.	Very low and highly variable protein content. Results depend critically on collection method (Schirmer strip vs. capillary), stimulation and reflex tearing, and on tear-film volume. Strong confounding by ocular-surface disease, contact-lens wear and topical medication, including preservatives. No reference intervals. Crucially, the relationship between ocular-surface and posterior-segment redox state is assumed rather than demonstrated.
Aqueous humour	Invasive; paracentesis, in practice only at the time of intraocular surgery.	Anterior segment; shares outflow with the trabecular meshwork; indirect for the retina.	MDA, GSH/GSSG, total antioxidant capacity, redox-related microRNAs, cytokines.	Directly informative for trabecular and anterior-segment redox biology in glaucoma; low protein content simplifies the matrix.	Sample volume of roughly 100–200 µL precludes replicate testing and limits multiplexing. Obtainable almost exclusively from surgical patients, that is, from advanced disease, which imposes a systematic spectrum bias. Blood or lens-material contamination. Because healthy eyes cannot ethically be sampled, “control” ranges derive from cataract-surgery patients, who are neither young nor redox-normal.
Vitreous humour	Invasive; vitrectomy or vitreous tap.	Immediately adjacent to the neuroretina.	Oxidative and inflammatory proteome, 4-HNE adducts, iron species.	The closest accessible fluid compartment to the retina; well suited to discovery proteomics.	Available only from eyes with vitreoretinal pathology requiring surgery. Regional and gel-versus-liquid heterogeneity means that the sampling site alters the result. Protein content varies by orders of magnitude with blood–retinal barrier breakdown, so concentrations are not comparable across disease states without normalization. Blood contamination is common.
Retinal tissue	Post-mortem or experimental only.	The target tissue itself.	8-OHdG/8-OHG, 4-HNE, TXNIP, iron and lipid-peroxidation indices, mitochondrial readouts.	The only matrix permitting cell-type and subcellular localisation, for example of nucleic-acid oxidation to RGC mitochondria.	Not available in living patients. Post-mortem interval, agonal state, hypoxia and fixation all generate artefactual oxidation, and the direction of that artefact is the same as the direction of the hypothesis. Cross-sectional by definition and confined to end-stage disease.
Blood (serum/plasma)	Minimally invasive; routine; freely repeatable.	Systemic; separated from the retina by the blood–retinal barrier.	MDA, F2-isoprostanes, protein carbonyls, 8-OHdG, GSH/GSSG, SOD, redox-related microRNAs.	The only matrix compatible with large longitudinal cohorts and with routine practice; broad assay availability; large existing biobanks.	Reflects whole-body redox load and has essentially no organ specificity. Strongly confounded by age, sex, smoking, diet and supplement use, exercise, renal and hepatic function, and comorbidity. Haemolysis inflates erythrocyte-derived analytes (SOD, GSH). MDA measured as thiobarbituric-acid-reactive substances is notoriously non-specific and is not interchangeable with chromatographic MDA. Pre-analytical handling (time to centrifugation, freeze–thaw cycles, storage temperature) materially alters results and is rarely reported.
Cerebrospinal fluid	Invasive; lumbar puncture.	A CNS compartment; the reference matrix for cerebral neurodegeneration.	F2-isoprostanes, protein carbonyls, 8-OHdG, oxidative and inflammatory metabolites, alongside Aβ42, p-tau and NfL.	The only fluid with an established, regulator-accepted neurodegeneration panel against which oxidative markers can be benchmarked.	Not repeatable at short intervals and poorly accepted for screening. A rostro-caudal concentration gradient makes results dependent on the volume fraction collected. Blood contamination. No oxidative analyte has approached the standardization achieved for Aβ42, p-tau and NfL, so oxidative CSF markers remain research assays in a matrix that otherwise sets the standard.
Retinal imaging (OCT/OCTA)	Non-invasive; repeatable within minutes.	In vivo and direct, of the target tissue.	RNFL and GCC thickness, macular volume, vessel density and perfusion metrics.	The only modality combining direct in vivo access to CNS tissue with quantitative, longitudinal, low-burden measurement; the natural anchor of the “eye as a window” concept.	Structural and vascular, not redox-specific: it reports the consequence of injury, not its mechanism. Strong confounding by refractive error and axial length, age, IOP, media opacity and comorbid ocular disease, all of which are more prevalent in the elderly populations of interest. Device-, segmentation-algorithm- and normative-database-dependent, which limits pooling across studies. Effect sizes are small relative to inter-individual variability.

**Table 6:** Route of administration, blood–retinal and blood–brain barrier penetration, and ocular exposure of the principal redox-active agents mapped in this review.

Agent (Class)	Routes Evaluated	Blood–Retinal/Blood–Brain Barrier Penetration	Reported Ocular Exposure	Principal Limitation for Clinical Translation	Refs
Coenzyme Q10 (mitochondrial electron carrier)	Oral; topical (vitamin E-TPGS-conjugated eyedrop); intravitreal (preclinical).	Poor after oral dosing. At 863 Da with an extremely high partition coefficient and negligible aqueous solubility, it is effectively unable to cross the blood-retinal or blood–brain barrier in useful amounts. The TPGS-conjugated eyedrop exists specifically to bypass this.	Oral dosing raises plasma concentrations with little measurable retinal exposure; the TPGS eyedrop achieves retinal levels in the diabetic rodent eye.	The QE3 trial of high-dose oral CoQ10 in early Parkinson disease was stopped for futility: a delivery failure that has been widely misread as target failure. Systemic dosing is the wrong route for this molecule; topical formulation is the informative test and has not been done clinically.	[78,178,179,180,189]
Idebenone (short-chain quinone analogue)	Oral.	Substantially better than CoQ10: at 338 Da with a short hydroxydecyl side chain it does reach the CNS and retina.	Detectable retinal and CNS exposure in humans.	Demonstrates that penetration is necessary but not sufficient. RHODOS, the only randomized trial of an antioxidant in a hereditary optic neuropathy, missed its primary endpoint despite adequate exposure; benefit was confined to a post hoc subgroup.	[190]
NRF2 activators: bardoxolone methyl, omaveloxolone (semisynthetic triterpenoids)	Oral (systemic).	Bardoxolone methyl distributes poorly to the CNS; omaveloxolone was optimized for CNS penetration and is approved for Friedreich ataxia.	Retinal target engagement demonstrated in the rat NAION model.	Systemic KEAP1–NRF2 activation is not tissue-selective and NRF2 is cytoprotective in malignant as well as healthy cells. Fluid retention and cardiac events terminated the BEACON trial of bardoxolone methyl. No ocular formulation exists, so the ocular data rest entirely on systemic dosing in rodents.	[125,181,182]
NACET (N-acetyl-L-cysteine ethyl ester)	Oral; eyedrop (preclinical).	Esterification confers the lipophilicity that N-acetylcysteine lacks, permitting cell and tissue entry followed by intracellular hydrolysis to cysteine.	Raises retinal glutathione in the mouse.	Preclinical only. Ester hydrolysis rates differ markedly between rodent and human plasma, so rodent exposure systematically over-predicts the human situation; this is the specific reason the parent compound failed.	[51]
NAD^+^ precursors (nicotinamide, nicotinamide riboside)	Oral.	Nicotinamide (122 Da) crosses both barriers readily; delivery is not the limiting step.	Raises systemic NAD^+^; retinal NAD^+^ elevation is inferred rather than measured in humans.	The best clinical evidence in glaucoma comes from small, short crossover studies using electrophysiological rather than structural or visual-field endpoints. Because delivery is not limiting, these trials are a genuine test of the hypothesis, which makes their modest effect sizes informative rather than dismissible.	[78,79,163]
Resveratrol and dietary polyphenols	Oral; topical.	Extensive first-pass glucuronidation and sulfation give oral bioavailability well below 1%; the parent compound scarcely reaches the retina.	The micromolar concentrations used in vitro are not attainable in retinal tissue in vivo.	The gap between the concentrations at which neuroprotection is demonstrated and those achievable in tissue is the single largest reason polyphenol neuroprotection has not translated, and it is a pharmacokinetic rather than a pharmacodynamic failure.	[42,120,186]
Molecular hydrogen	Inhalation; hydrogen-rich saline; eyedrops.	Freely diffusible; no barrier constraint whatsoever.	Reaches all ocular and cerebral compartments.	The converse problem: distribution is ideal but the proposed mechanism is contested, reactivity towards physiologically relevant oxidants is very low, and clearance is rapid.	[184]
TEMPOL and nitroxide hybrids	Systemic; topical (preclinical).	Small and membrane-permeant.	Ocular exposure demonstrated in rodents.	Rapid bioreduction to the inactive hydroxylamine in vivo shortens the effective half-life, and systemic hypotension is dose-limiting. Tissue concentration therefore correlates poorly with administered dose.	[183]
Manganese porphyrins (SOD mimetics)	Systemic; intravitreal (preclinical).	Cationic and hydrophilic; limited passive barrier transfer.	Ocular data limited.	Pro-oxidant behaviour at higher concentrations and in the presence of ascorbate gives a narrow therapeutic window, so under-dosing to achieve safety may preclude efficacy.	[47,73]
Ferroptosis inhibitors and iron chelators (ferrostatin-1, liproxstatin-1, deferiprone)	Intravitreal or intraperitoneal (preclinical); oral (deferiprone).	Ferrostatin-1 has poor metabolic stability and limited barrier transfer; deferiprone is orally active and does cross.	No ocular clinical data.	Systemic chelation carries real risk (agranulocytosis with deferiprone), and iron is essential to retinal metabolism, so the therapeutic index of retinal iron depletion is unknown. This is the most mechanistically promising family and the least tested.	[121,122,191]
MSC secretome; plasma rich in growth factors (PRGF) eyedrops	Topical; intravitreal; subretinal.	Not applicable: delivered directly to the target compartment, so the barrier is bypassed rather than crossed.	High local exposure.	Composition is undefined and donor-variable, with no release specification and no identified active principle; the regulatory pathway for a biological of variable composition is unclear.	[57,188,192]
OXR1 gene therapy	Subretinal or intravitreal AAV.	Not applicable: bypasses the barrier entirely.	Transduction of the target cells.	Irreversible, with vector immunogenicity and dose-limiting intraocular inflammation. RGC transduction from the vitreous remains inefficient in primates, so rodent efficacy does not predict human delivery.	[144]

## Data Availability

All the files relating to this research are available from the Corresponding Author, on reasonable request.

## Supplementary Materials

The following supporting information can be downloaded at: https://www.mdpi.com/article/10.3390/antiox15080948/s1, Table S1: Complete electronic search strategies used for the scoping review.

## Abbreviations

The following abbreviations are used in this manuscript:

Abbreviation	Full Term
4-HNE	4-Hydroxynonenal
8-OHdG	8-Hydroxy-2′-Deoxyguanosine
8-OHG	8-Hydroxyguanosine
ACSL4	Acyl-CoA Synthetase Long-chain Family Member 4
AD	Alzheimer’s Disease
ALS	Amyotrophic Lateral Sclerosis
AMD	Age-related Macular Degeneration
ARE	Antioxidant Response Element
CNS	Central Nervous System
CSF	Cerebrospinal Fluid
Drp1	Dynamin-related Protein 1
FSP1	Ferroptosis Suppressor Protein 1
GCC	Ganglion Cell Complex
GPX4	Glutathione Peroxidase 4
GSH	Reduced Glutathione
GSSG	Oxidised Glutathione
HD	Huntington’s Disease
IOP	Intraocular Pressure
JBI	Joanna Briggs Institute
KEAP1	Kelch-like ECH-associated Protein 1
LHON	Leber Hereditary Optic Neuropathy
LPCAT3	Lysophosphatidylcholine Acyltransferase 3
MCI	Mild Cognitive Impairment
MDA	Malondialdehyde
MS	Multiple Sclerosis
mtDNA	Mitochondrial DNA
NAD^+^/NADH	Nicotinamide Adenine Dinucleotide (Oxidized/Reduced)
NF-κB	Nuclear Factor Kappa B
NLRP3	NOD-like Receptor Family Pyrin Domain-containing 3
NOS	Nitric Oxide Synthase
NOX/NOX4	NADPH Oxidase/NADPH Oxidase 4
NRF2	Nuclear Factor Erythroid 2-related Factor 2
OCT/OCTA	Optical Coherence Tomography/Angiography
OHT	Ocular Hypertension
OPTN	Optineurin
OXR1	Oxidative Stress Resistance 1
OXPHOS	Oxidative Phosphorylation
PCC	Population-Concept-Context
PD	Parkinson’s Disease
POAG	Primary Open-Angle Glaucoma
PRISMA-ScR	Preferred Reporting Items for Systematic Reviews and Meta-Analyses Extension for Scoping Reviews
PUFA	Polyunsaturated Fatty Acid
RGCs	Retinal Ganglion Cells
RNFL	Retinal Nerve Fibre Layer
ROS/RNS	Reactive Oxygen/Nitrogen Species
RPE	Retinal Pigment Epithelium
SOD	Superoxide Dismutase
TNF-α	Tumour Necrosis Factor alpha
TRPV4	Transient Receptor Potential Vanilloid 4
TXNIP	Thioredoxin-interacting Protein
