# Supplementary material for: The Eye as a Window to Neurodegeneration: Oxidative Stress, Optic Nerve Vulnerability, and Retinal Biomarkers—A Scoping Review
Source: Antioxidants (Basel). 2026 Jul 30;15(8):948. doi: 10.3390/antiox15080948 (PMC13509633; doi:10.3390/antiox15080948)
Supplement: Supplementary file 1 [file antioxidants-15-00948-s001.zip › antioxidants-4434985-supplementary.pdf]

Supplementary Table S1. Complete electronic search strategies used for the scoping review.

Database	Search strategy
**PubMed/MEDLINE**	((“oxidative stress”[MeSH Terms] OR “oxidative stress”[Title/Abstract] OR “reactive oxygen species”[Title/Abstract] OR ROS[Title/Abstract] OR “reactive nitrogen species”[Title/Abstract] OR RNS[Title/Abstract] OR redox[Title/Abstract] OR ferroptosis[Title/Abstract] OR “mitochondrial dysfunction”[Title/Abstract]) AND (“neurodegenerative diseases”[MeSH Terms] OR neurodegeneration[Title/Abstract] OR “Alzheimer disease”[MeSH Terms] OR “Alzheimer disease”[Title/Abstract] OR “Parkinson disease”[MeSH Terms] OR “Parkinson disease”[Title/Abstract] OR “amyotrophic lateral sclerosis”[MeSH Terms] OR “amyotrophic lateral sclerosis”[Title/Abstract] OR ALS[Title/Abstract] OR “Huntington disease”[MeSH Terms] OR “Huntington disease”[Title/Abstract] OR dementia[Title/Abstract]) AND (“optic nerve”[MeSH Terms] OR “optic nerve”[Title/Abstract] OR retina[MeSH Terms] OR retina[Title/Abstract] OR “retinal ganglion cell”[Title/Abstract] OR “retinal ganglion cells”[Title/Abstract] OR glaucoma[MeSH Terms] OR glaucoma[Title/Abstract] OR “optic neuropathy”[Title/Abstract] OR “optic neuropathies”[Title/Abstract] OR vision[Title/Abstract] OR “visual dysfunction”[Title/Abstract] OR neuro-ophthalmology[Title/Abstract]))
**Embase**	(‘oxidative stress’/exp OR ‘oxidative stress’:ti,ab OR ‘reactive oxygen species’:ti,ab OR ROS:ti,ab OR ‘reactive nitrogen species’:ti,ab OR RNS:ti,ab OR redox:ti,ab OR ferroptosis:ti,ab OR ‘mitochondrial dysfunction’:ti,ab) AND (‘neurodegenerative disease’/exp OR neurodegeneration:ti,ab OR ‘Alzheimer disease’/exp OR ‘Alzheimer disease’:ti,ab OR ‘Parkinson disease’/exp OR ‘Parkinson disease’:ti,ab OR ‘amyotrophic lateral sclerosis’/exp OR ‘amyotrophic lateral sclerosis’:ti,ab OR ALS:ti,ab OR ‘Huntington disease’/exp OR ‘Huntington disease’:ti,ab OR dementia:ti,ab) AND (‘optic nerve’/exp OR ‘optic nerve’:ti,ab OR ‘retina’/exp OR retina:ti,ab OR ‘retinal ganglion cell’:ti,ab OR ‘retinal ganglion cells’:ti,ab OR ‘glaucoma’/exp OR glaucoma:ti,ab OR ‘optic neuropathy’:ti,ab OR ‘optic neuropathies’:ti,ab OR vision:ti,ab OR ‘visual dysfunction’:ti,ab OR ‘neuro ophthalmology’:ti,ab OR neuro-ophthalmology:ti,ab)
**Scopus**	TITLE-ABS-KEY (“oxidative stress” OR “reactive oxygen species” OR ROS OR “reactive nitrogen species” OR RNS OR redox OR ferroptosis OR “mitochondrial dysfunction”) AND TITLE-ABS-KEY (neurodegeneration OR “neurodegenerative disease” OR “neurodegenerative diseases” OR “Alzheimer disease” OR “Parkinson disease” OR “amyotrophic lateral sclerosis” OR ALS OR “Huntington disease” OR dementia) AND TITLE-ABS-KEY (“optic nerve” OR retina OR “retinal ganglion cell” OR “retinal ganglion cells” OR glaucoma OR “optic neuropathy” OR “optic neuropathies” OR vision OR “visual dysfunction” OR neuro-ophthalmology OR “neuro ophthalmology”)
**Web of Science Core Collection**	TS=(“oxidative stress” OR “reactive oxygen species” OR ROS OR “reactive nitrogen species” OR RNS OR redox OR ferroptosis OR “mitochondrial dysfunction”) AND TS=(neurodegeneration OR “neurodegenerative disease” OR “neurodegenerative diseases” OR “Alzheimer disease” OR “Parkinson disease” OR “amyotrophic lateral sclerosis” OR ALS OR “Huntington disease” OR

	dementia) AND TS=("optic nerve" OR retina OR "retinal ganglion cell" OR "retinal ganglion cells" OR glaucoma OR "optic neuropathy" OR "optic neuropathies" OR vision OR "visual dysfunction" OR neuro-ophthalmology OR "neuro ophthalmology")
